# Repurposing the lineage-determining transcription factor Atoh1 without redistributing its genomic binding sites

**DOI:** 10.3389/fcell.2022.1016367

**Published:** 2022-11-07

**Authors:** Aida Costa, Lynn M. Powell, Mattias Malaguti, Abdenour Soufi, Sally Lowell, Andrew P. Jarman

**Affiliations:** ^1^ Centre for Discovery Brain Sciences, Edinburgh Medical School, University of Edinburgh, Edinburgh, United Kingdom; ^2^ MRC Centre for Regenerative Medicine, Institute for Stem Cell Research, School of Biological Sciences, University of Edinburgh, Edinburgh, United Kingdom

**Keywords:** hair cell, Atoh1, pioneer factors, cochlea, Gfi1, Pou4f3, inner ear

## Abstract

Although the lineage-determining ability of transcription factors is often modulated according to cellular context, the mechanisms by which such switching occurs are not well known. Using a transcriptional programming model, we found that Atoh1 is repurposed from a neuronal to an inner ear hair cell (HC) determinant by the combined activities of Gfi1 and Pou4f3. In this process, Atoh1 maintains its regulation of neuronal genes but gains ability to regulate HC genes. Pou4f3 enables Atoh1 access to genomic locations controlling the expression of sensory (including HC) genes, but Atoh1 + Pou4f3 are not sufficient for HC differentiation. Gfi1 is key to the Atoh1-induced lineage switch, but surprisingly does not alter Atoh1’s binding profile. Gfi1 acts in two divergent ways. It represses the induction by Atoh1 of genes that antagonise HC differentiation, a function in keeping with its well-known repressor role in haematopoiesis. Remarkably, we find that Gfi1 also acts as a co-activator: it binds directly to Atoh1 at existing target genes to enhance its activity. These findings highlight the diversity of mechanisms by which one TF can redirect the activity of another to enable combinatorial control of cell identity.

## 1 Introduction

During development, lineage commitment often relies on key transcription factors (TFs) that are necessary and sufficient to regulate the gene expression programme specific to a particular cell fate in a particular context. Such TFs, sometimes referred to as master regulators, include basic helix-loop-helix (bHLH) domain TFs such as MyoD, which determines muscle fate (Tapscott et al., 1988), and proneural factors, which determine neural fates (Masserdotti et al., 2016). Often, however, a TF can drive the specification of several seemingly unrelated cell types. How one TF can drive the formation of diverse cell types is a central question in developmental biology. Part of the answer is that master regulator TFs work in partnership with other TFs, with lineages defined by combinatorial codes of TFs. However, the mechanisms by which TFs modulate each other’s function are poorly understood. Recent ChIP-seq experiments have demonstrated that in at least some cases the same TF is targeted to different sites in the genome to determine alternative cellular identities according to composition of the combinatorial TF code. It remains an open question whether there are also cases where a TF defines distinct lineages while remaining at the same genome locations.

It can be difficult to examine to what extent a particular TF is responsible for modifying DNA binding of another TF. Numerous cellular variables such as DNA accessibility, epigenetic landscape, TF cooperativity, cofactors, and enhancer activity influence the genome occupancy of TFs, making it difficult to unpick the relative influence of each of these variables when comparing two different lineage specification events. The advent of *in vitro* cell fate reprogramming has demonstrated that overexpression of specific TF combinations can be sufficient to drive specification towards diverse cell types in the absence of other context-specific variables (Pfisterer et al., 2011; Zhao et al., 2015; Masserdotti et al., 2016; Nakamori et al., 2017). Therefore, cellular reprogramming provides a controlled system in which to investigate how TFs cooperate and modulate each other’s functions for cell fate specificity in the absence of other differences in cellular context (Aydin et al., 2019).

The bHLH-domain TF Atoh1 is an example of a cell lineage regulator that promotes the specification of diverse cell types in diverse tissue contexts (Jarman and Groves 2013). Atoh1-dependent cell types include: 1) sensory hair cells (HCs) located in the inner ear (Bermingham et al., 1999; Chen et al., 2002; Pan et al., 2011) 2) a subset of neurons present in spinal cord and cerebellum (Klisch et al., 2011; Lai et al., 2011) 3) Merkel cells of the skin (Maricich et al., 2009), and 4) secretory cells in the intestine (Yang et al., 2001). Atoh1 function in HC formation is of particular interest because of its potential use in gene therapy to promote HC regeneration in sensorineural deafness. This arises from the finding that Atoh1 is not only necessary but also sufficient for HC formation in rodent models (Bermingham et al., 1999; Zheng and Gao 2000; Jarman and Groves 2013). However, *in vivo* HC regeneration resulting from experimentally supplied Atoh1 is very inefficient, but the factors limiting Atoh1’s function are poorly known (Costa et al., 2015).

There are several obstacles to understanding how Atoh1 specifically drives HC differentiation in the inner ear even though it has the ability to specify other cell types in other contexts. First, the spatial and temporal heterogeneity of embryonic tissues *in vivo* are difficult to dissect. Second, identifying *in vivo* binding sites by ChIP-seq requires large numbers of Atoh1-expressing cells. Atoh1 ChIP-seq analysis has been achieved in the context of cerebellar granule neurons (Lai et al., 2011) and intestinal crypt cells (Kim et al., 2014) but it is difficult in tissues where Atoh1-expressing cells are scarce such as the inner ear. For these reasons, the genome-wide binding occupancy profile of Atoh1 in HCs is yet to be determined.

Previously, we established the first direct programming strategy for generating a reliable supply of HC-like cells (induced HCs, iHCs) from mouse embryonic stem cells (mESCs) (Costa et al., 2015). Although forced expression of Atoh1 alone promoted neuronal differentiation, co-delivery of Gfi1 and Pou4f3 with Atoh1 promoted remarkably robust and efficient iHC differentiation. This suggests that Pou4f3 and Gfi1 can convert Atoh1 from a neuronal determinant to an HC determinant, in keeping with the fact that Pou4f3 and Gfi1 are both vital for HC survival and differentiation *in vivo* (Wallis et al., 2003; Hertzano et al., 2004). However, it is not known how these two TFs modulate Atoh1’s function. Based on our current understanding, an attractive hypothesis would be that Pou4f3 and Gfi1 repress Atoh1’s neuronal transcriptional programme by simply redistributing Atoh1 binding away from neuronal genomic regulatory regions to new regions that control the expression of HC genes.

In this report, we used our TF programming strategy combined with genome-wide approaches (ChIP-seq and RNA-seq) to determine the effects of Gfi1 and Pou4f3 on Atoh1’s DNA binding and transcriptional activities. We demonstrate that Atoh1 can activate a neuronal programme in mESCs and confirm that Gfi1 and Pou4f3 convert Atoh1 to a sensory HC determinant. Surprisingly, however, Gfi1 and Pou4f3 do not repress Atoh1’s neuronal programme during this process, but instead confer on Atoh1 an additional capacity for HC gene regulation. Pou4f3 and Gfi1 achieve this in different ways. Pou4f3 recruits Atoh1 to new genomic sites, but by itself this results in the activation of a generic sensory program and reinforcement of neuronal differentiation. Unlocking the HC programme requires Gfi1, but it does not act by altering the DNA binding profiles of Atoh1 or Pou4f3. Instead, Gfi1 has two functions. First, it represses inappropriate gene expression, consistent with previously described roles in hematopoiesis (van der Meer et al., 2010). Second, Gfi1 interacts with Atoh1 at existing sites as a potent transcriptional co-activator to augment both neuronal and HC-specific sensory programs. Overall, these findings demonstrate how repurposing lineage determining TFs is not necessarily achieved through major change in their binding profiles but can be accomplished in part through more subtle effects such as transcriptional amplification of pre-existing binding sites. Therapeutically, these results suggest new avenues to overcome Atoh1’s limitations in promoting HC regeneration.

## 2 Materials and methods

### 2.1 Generation, growth and differentiation of doxycycline-inducible mESC lines

**FIGURE 1:**
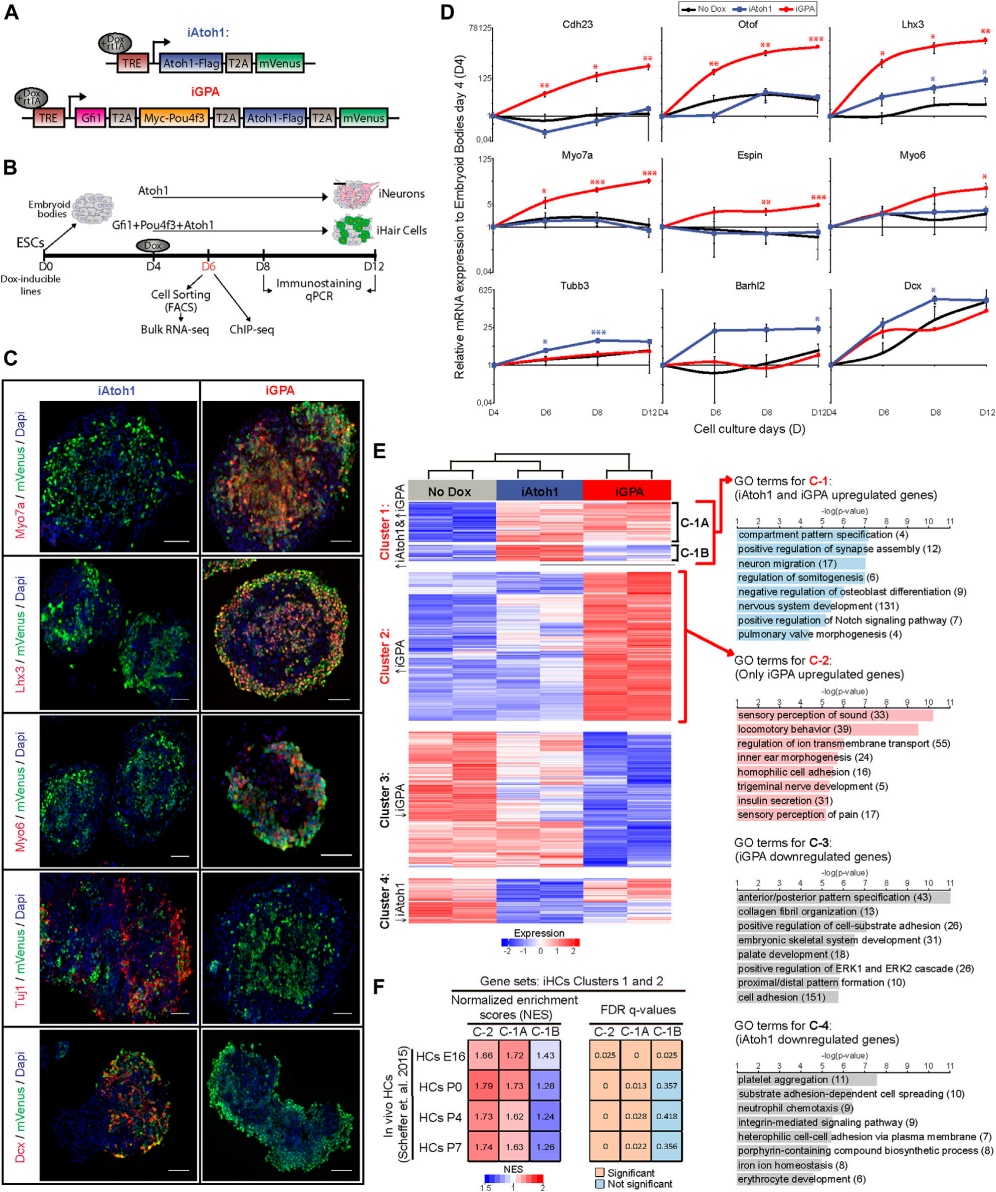
Atoh1 drives neuronal differentiation while Gfi1 + Pou4f3 + Atoh1 drives HC differentiation. **(A)** Dox inducible tagged TF constructs used in this study for iAtoh1 and iGPA lines. T2A, 2A self-cleaving peptide derived from thosea asigna virus, TRE, tetracycline responsive element, rtTA, reverse tetracycline transactivator. **(B)** Schematic differentiation protocol for TF induction timeline in EBs and analyses time-points. **(C)** Representative images obtained from immunostaining of HC (Myo7a, Lhx3, and Myo6) and neuronal (Tuj1, Dcx) markers (red) in induced EBs at day 8 after 4 days of Dox treatment. mVenus (green) indicates cells overexpressing the transgenic TF combination and nucleus are labelled by DAPI (blue). Scale bar, 50 µm. **(D)** qRT-PCR analyses for HC markers (Cdh23, Otof, Lhx3, Myo7a, Espin, and Myo6) and neuronal markers (Tubb3, Barhl2, and Dcx) in EBs derived from iAtoh1 and iGPA ESC lines after 2, 4, and 8 days of Dox treatment (2 ug/ml of Dox was added at day 4 of EB culture and analyses were carried out at day 6, 8, and 12 of EB culture, respectively). Relative expression of each transcript is presented as fold change normalized to the mean of EBs at culture day 4 before Dox treatments. Results are mean ± s.e.m. *p < 0.1, **p < 0.01, ***p < 0.001 compared to no Dox control (*n* = 3, biological replicates). **(E)** Heatmap displaying the gene expression levels for 2,818 unique genes consider to be differentially expressed in both iAtoh1 and iGPA relative to uninduced EBs. These genes were clustered accordantly to their similar expression pattern across the three different conditions: No Dox, iAtoh1, and iGPA. The clustering analyses identified four major gene clusters, C-1 represents mainly genes enriched in both iAtoh1 and iGPA, C-2 shows genes enriched only in iGPA, C-3 and C-4 highlights the genes downregulated in iAtoh1 and iGPA, respectively; C-1 was further divided into two sub-clusters: C-1A contains 363 genes enriched in both iAtoh1 and iGPA and C-1B contains 148 genes that are enriched mainly in iAtoh1 but not in iGPA. GO analyses was performed for each of the 4 major gene clusters and the most significant GO terms for biological process are shown as well as the number of genes included within each GO term. **(F)** Gene set enrichment analysis (GSEA) performed using a published *in vivo* mouse HC transcriptome dataset (GSE60019) containing multiple HC differentiation time-points; Embryonic day 16 (HC E16) and several postnatal periods (P0, P4, and P7). The gene sets analysed here were defined by the above gene clusters C-1A, C-1B, and C-2. The tables show the normalised enrichment scores (NES) and FDR q-values obtained for each cluster C-1A, C-1B, and C-2 across early and later stages of HC differentiation.

For the generation of the dox-inducible mESC lines expressing different combinations of Atoh1 (A), Pou4f3 (P) and Gfi1 (G) (iAtoh1, iPou4f3, iGfi1, iG+P, iG+A, iP+A and iGPA), the following plasmids were constructed following the procedures described below (Figure 1A). All primers are given in Supplementary Table S1. All plasmids and full sequences are available upon request:

#### 2.1.1 p2Lox-Atoh1F-Venus

A 3xFlag tag was added to the 3′ end of the murine Atoh1 coding sequence by PCR amplification with GPAPlox vector (Costa et al., 2015) as a template. The PCR product was cloned into a 2AP-mVenusNLSPlox vector (Costa et al., 2015 unpublished plasmid). Next, the entire sequence the Atoh1-3xFlag-2AP-mVenusNLS was excised and cloned into the p2lox plasmid (Addgene #34635) (Iacovino et al., 2011).

#### 2.1.2 p2Lox-MPou4f3-Venus

A 3xMyc was added to the 5′ end of the murine Pou4f3 coding sequence by PCR amplification with GPAPlox as a template. The PCR product was cloned into a 2AP-mVenusNLSPlox vector and them, the entire sequence the 3xMyc-Pou4f3-2AP-mVenusNLS was excised and cloned into the p2lox.

#### 2.1.3 p2Lox-Gfi1-Venus

Murine *Gfi1* ORF was PCR amplified from the GPAPlox plasmid. The PCR product was cloned into a 2AP-mVenusNLSPlox vector and them, the entire sequence the Gfi1-2AP-mVenusNLS was excised and cloned into the p2lox.

#### 2.1.4 p2Lox-Gfi1 + MPou4f3-Venus

Murine *Gfi1* ORF was PCR amplified from the GPAPlox plasmid. The PCR product was cloned into the p2Lox-MPou4f3-Venus plasmid.

#### 2.1.5 p2Lox-Gfi1 + Atoh1F-Venus

The 3xFlag tag Atoh1 sequence was PCR amplified using p2Lox-Atoh1F-Venus as a template. This PCR product was inserted into the p2Lox-Gfi1 + MPou4f3-Venus plasmid by excising first the 3xMyc-Pou4f3 coding sequence and replacing it with 3xFlag-Atoh1.

#### 2.1.6 p2Lox-MPou4f3 + Atoh1F-Venus

The 3xMyc tag was added to the 5′ end of the Pou4f3 coding sequence by PCR amplification and the GPAPlox as a template. The PCR product was cloned into the Atoh1-3xFlag-2AP-mVenusNLSPlox vector and the entire sequence the Myc3x-Pou4f3-2AP-Atoh1-3xFlag-2AP-mVenusNLS was excised and cloned into the p2lox.

#### 2.1.7 p2Lox-Gfi1 + MPou4f3 + Atoh1F-Venus

*Gfi1* ORF was PCR amplified from the GPAPlox plasmid. The PCR product was cloned into the Myc3x-Pou4f3-2AP-Atoh1-3xFlag-2AP-mVenusNLSPlox vector and the entire sequence the Gfi1-2AP-Myc3x-Pou4f3-2AP-Atoh1-3xFlag-2AP-mVenusNLS was excised and cloned into the p2lox.

All plasmids were confirmed by sequencing. Plasmids were nucleofected (Amaxa 4D-Nucleofector, Lonza) into A2Lox cells (gift from Prof. Lesley Forrester) as described previously (Iacovino et al., 2011; Iacovino et al., 2014) Cells were subsequently plated on neomycin-resistant, gamma irradiated MEF feeder cells (Stem Cell Technology, #00323). Two days after nucleofection, the recombined cells were selected by using DMEM medium supplemented with 350 µg/ml G418 (InvivoGen). Individual colonies were picked after 1 week (Iacovino et al., 2014).

### 2.2 mESC maintenance and embryoid body differentiation

mESCs were routinely grown on top of mitotically inactivated mouse embryonic fibroblasts (MEFs) at 37°C in a 5% CO_2_ incubator in Dulbecco’s Modified Eagle’s Medium (DMEM), supplemented with 10% fetal bovine serum (FBS) (ES-qualified), 100 U/ml human leukemia inhibitory factor (LIF), 2 mM L-Glutamine, 1 mM sodium pyruvate, 1 mM 2-mercaptoethanol, 1% of penicillin-streptomycin and 1x non-essential amino acids (all from Life Technologies). Cells were passaged every other day, at constant plating density of 3 × 10^4^ cells/cm^2^.

For EB differentiation, MEFs were first depleted from the mESCs by using 0.25% trypsin-EDTA (Life Technologies) to dissociate MEFs-coated mESCs culture dishes into a single cell suspension which was incubated on gelatin-coated (0.1%) dishes for 45 min at 37°C. After removing the medium containing unattached mESCs suspension, mESCs were seeded on 60-mm bacterial-grade Petri dishes at 3 × 10^4^ cells/cm^2^ in the same DMEM medium, but in the absence of LIF. EBs formed within 24 h, and medium was changed every 2 days. Supplementation with 2 µg/ml doxycycline (diluted in sterile PBS and filtered through a 0.2 μm filter unit) (Sigma-Aldrich) and/or 1 µM retinoic acid (RA) (diluted in 0.01% DMSO) (Sigma-Aldrich) were initiated at day 4 and maintained until the required time point for analysis (48 h, 4 days or day 8 after doxycycline treatment).

MEFs were derived from wild-type E14, 5 mouse embryos following isolation and growth procedures described in (Garfield 2010). Mitotically inactivated MEFs were obtained using 50 Gy of gamma irradiation.

### 2.3 Immunocytochemistry and imaging

EBs (6, 8, and 12 days old) were fixed with 1% paraformaldehyde during 15 min at room temperature (RT). Fixed EBs were cryoprotected in 15% sucrose in PBS, embedded in a solution containing 7.5% gelatine (Sigma-Aldrich) and 15% sucrose in PBS, frozen and cryosectioned (8–10 μm). EB sections were immersed in PBS at 37°C until gelatine was completely dissolved, and then processed for immunocytochemistry. Sections were blocked with 10% FBS and 0.05% Tween in PBS for 1 h, followed by incubation overnight with the following primary antibodies: Anti-MyoVIIa (1:400, HPA028918, Sigma), Anti-MyoVI (1:50, 25-6791, Proteus Biosciences) Anti-Pou4f3 (1:50, HPA038215, Sigma), Anti-Espin (1:1,000, gift of A. J. Hudspeth), Anti-Gfi1 (1:2,000, gift of Hugo Bellen), Anti-Gfi1 (ab21061 or ab290, Abcam), Anti-Lhx3 (1:100, ab14555, Abcam), Anti-Tuj1 (1:500, MMS-435P, Covance), Anti-GFP (1:500, ab13970, Abcam), Anti-Doublecortin (1:1,000, AB2253, Merck Millipore). Sections were washed 3 times in PBS followed by incubation for 1 h at RT with AlexaFluor-conjugated secondary antibodies (1:400, Molecular Probes) and 0.15% DAPI (Sigma-Aldrich). Slides where then mounted with prolong gold (Life technologies). Fluorescent images of fixed sections were captured with Widefield Zeiss observer or using a Leica TCS SP8 Confocal 4 Detectors. All digital images were formatted with Adobe Photoshop CS and ImageJ.

### 2.4 Flow cytometry

For live cell analyses to measure Venus expression in all dox-inducible lines, EBs were dissociated and re-suspended in PBS with 2% FBS after 48 h, 4 and 8 days of dox treatment. All cells were analysed using FACSCalibur (BD Biosciences). Data were analysed with FlowJo software (Tree Star).

### 2.5 RNA extraction and real-time quantitative PCR

EBs (6, 8, and 12 days old) were dissociated and 106 cells were used to extract total RNA following manufacturer’s instructions of the Absolutely RNA Miniprep Kit (#400800, Agilent Technologies). cDNA was synthesised with 200 ng-1 µg of total RNA using M-MLV Reverse Transcriptase (Invitrogen) and random hexamers. Quantitative PCR was performed using Light Cycler 480 SYBR Green I Master Mix (Roche) in a LightCycler 480 II Instrument.

Primers were designed using NetPrimer program and PCR products were confirmed by proper melting curves and agarose gel electrophoresis. Values for each gene were normalized to the expression values of *Sdha* and expressed as mean ± s.e.m. (of at least three replicates) relative to control untreated samples (without Dox). The primer pairs sequences are described in Supplementary Table S1.

### 2.6 RNA sequencing

Total RNA was extracted from untreated EBs at day 6, and from FACS sorted EBs at day 6 previously treated with Dox for 48 h. Cell sorting of Venus + populations were done on a FACS Aria cell sorter (Becton Dickinson). RNA concentration and purity was determined by spectrophotometry and integrity was confirmed using a High Sensitivity RNA ScreenTape system on an Agilent 2200 TapeStation. RNA-seq libraries were generated with 1ug of total RNA using KAPA mRNA HyperPrep Kit (KK8580, Kapa Biosystems) following manufacturer’s instructions. RNA-seq libraries were pooled at equal concentration and sequenced with Edinburgh Genomics on the Illumina HiSeq 4000 platform. Two biological replicates per condition were processed.

Sequencing quality of the raw data was checked using FastQC (Andrews 2010) and MultiQC (Ewels et al., 2016). Contaminant adapters were trimmed using cutadapt (Martin 2011). The trimmed reads were pseudo-aligned to cDNA sequences from UCSC’s knownGene transcriptome (mm9 assembly) using Kallisto with default settings (Bray et al., 2016). For differential expression analyses, transcript abundances were imported into R and summarised to the gene-level using the tximport workflow (Soneson et al., 2015). Significantly differentially expressed genes (FDR < 0.05 and log2 fold change > 1.5) were identified using the standard DESeq2 package in Bioconductor (Love et al., 2014). The clustering patterns of genes were assessed based on a matrix of the mean of biological replicate samples. The matrix was clustered by use of the pam() function in R. Heatmaps visualizations containing mean normalized counts of each cross-classified gene group were generated using Complex Heatmaps package in Bioconductor (Gu et al., 2016). Lists of genes were analysed for Gene Ontology (GO) term enrichment using topGO package in Bioconductor (Alexa and Rahnenfuhrer 2019).

#### 2.6.1 Gene set enrichment analysis

*In vivo* hair cell transcriptomes obtained from mouse vestibular and cochlea tissue at embryonic (E16) and postnatal stages (P0, P4 and P7) were downloaded from GEO database, accession number GSE60019. Raw data (FASTQ files) was processed using the same RNA-seq analyses pipeline described above to generate a normalised matrix counts which was imported to GSEA software (Mootha et al., 2003; Subramanian et al., 2005). Defined gene set determined by the above described clustering analyses were also uploaded into the GSEA software to identify statistically significant enrichments between hair cells and non-sensory cell types of the inner ear.

### 2.7 Chromatin immunoprecipitation

ChIP-seq experiments were performed by adapting and modifying protocols described in (Ford et al., 2014; Ruetz et al., 2017). Six days old EBs treated with 2 µg/ml doxycycline at day 4 were dissociated using 0.25% trypsin-EDTA (Invitrogen) in PBS and around 15 × 106–20 × 106 of cells were aliquoted into 15 ml falcon tubes for fixation. Cells in each aliquot were fixed at room temperature for 12 min on 1% of formaldehyde solution (37% formaldehyde, Sigma-Aldrich). Formaldehyde was quenched with glycine (final 0.125 M) for 5 min at room temperature. Samples where then washed once with cold PBS containing protease inhibitors (complete protease inhibitor cocktail, Roche). After, cells were centrifuged at 500 g and pellets were snap frozen on dry ice followed by storage at −80°C until further use. The pellet for each sample (∼20 × 106 of cells) was thawed and nuclei were isolated by re-suspension in lysis buffer (5 mM Pipes pH 8.0, 85 mM KCl, 1% NP40) with fresh protease inhibitors (cOmplete protease inhibitor cocktail, Roche) for 20 min on ice followed by brief vortexing and centrifugation at 500 g for 10 min at 4°C. The nuclear pellet was re-suspended in 300 ul IP buffer (0.5% SDS, 1% Triton, 2 mM EDTA, 20 mM Tris-HCl pH 8.0, 150 mM NaCl, fresh protease inhibitors) and sheared by sonication (BioRuptor Pico, Diagenode) down to ∼200–300 bp fragments using a total of 5–14 cycles of: 10 pulses of sonication on the ‘‘high’’ setting, each followed by 30 s off. Samples were then diluted in IP buffer without SDS, then 10 mg of the following antibodies was added for overnight incubation at 4°C on a rotator: Monoclonal anti-flag M2 antibody (F1804, Sigma); Myc tag antibody-ChIP Grade (ab9132, Abcam); Anti-Gfi1 antibody (ab21061, Abcam). Dynabeads-ProteinG (Life Technologies) were blocked for 1-h in 0.5% BSA in IP buffer with protease inhibitors, prior to incubation with the sonicated antibody bound chromatin suspensions. Bead-chromatin complexes were then serially washed for 5 min each with the following solutions: IP buffer (150 mM NaCl), followed by IP buffer with high salt concentration (500 mM NaCl), then 1 wash with washing-buffer (10 mM Tris-HCl pH 8.0, 0.25 M LiCl, 0.5% NP40, 0.5% Na-Deoxycholate, 1 mM EDTA), followed by two washes with TE (10 mM Tris, 1 mM EDTA). The DNA-protein complex was then eluted from beads by incubation with 100 ml of elution buffer (1% SDS, 10 mM EDTA, 50 mM Tris-HCl pH 8.0) for 30 min at 65°C, vortexing every 10 min, followed by magnet extraction of beads. The beads were re-washed with 150 ml TE with 1% SDS, magnet extracted, and the TE with remaining DNA solution was added to the eluted samples, followed by 65°C overnight incubation to reverse crosslink the DNA-protein complexes. The dissociated DNA and protein solution was then treated with 4U of proteinase K (NEB) at 37°C for 2 h. DNA was isolated with SeraMag beads, using 450 ml SeraMag bead solution and 225 ml of 30% PEG in 1.25 M NaCl (Rohland and Reich 2012). After bead purification, DNA was re-suspended in 30 µl of ddH2O (DNase/RNase free) and for each ChIP sample, DNA concentration was estimated using Qubit fluorometric quantification (Invitrogen) before being stored at 80°C.

### 2.8 ChIP-seq: Library preparation

Eight–Ten ng of ChIP DNA was used for each sample. DNA ends were blunted by treatment with 5 µl T4 DNA ligase buffer (NEB), 2 µl 10 mM dNTP’s, 0.5 µl end repair mix (0.72 U T4 DNA polymerase, 0.24 U Klenow Fragment, 2.4 U T4 DNA Polynucleotide Kinase), up to 50 µl with ddH2O (DNase/RNase free). Samples were incubated for 30 min at 20°C, and then purified with addition of 50 ul SeraMag bead solution and 50 µl 30% PEG solution (in 1.25 M NaCl). DNA was eluted in 16.5 ul of TE/10 (10 mM TrisCl pH 8.0, 0.1 mM EDTA). DNA was then A-tailed at the 3′ ends by treating the eluate with 2 µl 10X NEB Buffer 2, 1 µl 4 mM dATP, 0.5 µl Klenow 3′ to 5′ exonuclease minus (NEB) in a total volume of 20 µl. Reaction was incubated at 37°C for 30 min. Before adapter ligation, TruSeq adapters (Illumina) were first annealed by re-suspending each adapter at 100 mM (in 10 mM Tris-HCl pH7.8, 0.1 mM EDTA pH 8.0, 50 mM NaCl) and mixing them at a 1:1 ratio. Adaptor annealing occurred using a program of 2 min at 95°C, 70 cycles of 30 s (95°C decreasing by 1°C each cycle). Annealed adaptors were diluted 1:200 (0.25 mM final) and then 1 µl was used for ligation reaction containing 20 µl of the A-tailed DNA, 1.5 µl Quick Ligase (2,000 U/ml NEB) and 2.5 µl of H_2_O. The reaction was incubated 20 min at room temperature and stopped by addition of 5 µl of 0.5 M EDTA pH 8.0. Next, DNA was purified using 50 µl SeraMag bead mix and 50 µl 30% PEG (in 1.25 M NaCl) and eluted in 15.5 µl of TE/10. Next, the adapter ligated DNA was amplified using the following protocol: 15 µl adaptor-ligated DNA, 1 µl TruSeq primer cocktail (0.25 mM), 15 µl 2X Kapa HiFi HotStart ready mix. The Libraries were amplified with the following protocol: 45 s at 98°C, 5 cycles of (15 s at 98°C, 30 s at 63°C, 30 s at 72°C), 1 min of 72°C, hold at 4°C. After pre-amplification of the library, a size selection step was performed to obtain DNA fragments between 300 and 500 bp using the following procedure: ×0.9 beads were added to ChIP DNA After 15 min incubation and 10 min magnet extraction, the supernatant was transferred to new tube and beads discarded. Then ×0.2 beads were added to the solution, incubated 15 min followed by 10 min magnet extraction and beads re-suspension in 11.5 µl TE/10. 11 µl of pre-amplified and size selected DNA was further amplified with 1 µl TruSeq primer cocktail, 20 µl 2X Kapa HiFi HotStart ready mix, and 8 µl H2O using a PCR program of 45 s at 98°C, 9-11 cycles of (15 s 98°C, 30 s 63°C, 30 s 72°C), 1-min 72°C. After amplification, the DNA was isolated using SeraMag bead purification. Libraries were assessed using Agilent High Sensitivity D1000 ScreenTape System on an Agilent 2200 TapeStation. Finally, libraries were pooled at equal concentration and sequenced with Edinburgh Genomics on the Illumina HiSeq 4000 platform. Two biological replicates were processed for all conditions except Gfi1, for which only a single useable library was obtained due to technical limitations of the antibody.

### 2.9 ChIP-seq: Data analyses

Reads quality was checked using FastQC (Andrews, 2010) and MultiQC (Ewels et al., 2016). Illumina contaminant adapter sequences were removed using Trimmomatic (Bolger et al., 2014). Reads were aligned to the UCSC mm9 assembly of the mouse genome using Bowtie 2 with the very-sensitive option (Langmead and Salzberg 2012). For downstream analysis, PCR duplicates reads were removed using Picard Tool’s MarkDuplicates command (Broad Institute) and were filtered for MAPQ ≥ 40 using SAMtools (Li et al., 2009). Reads aligned to ENCODE blacklist regions (Consortium 2012) were removed using BEDTools (Quinlan and Hall 2010). Unmapped, secondary, and supplemental read alignments were filtered using SAMtools. Peak calling was performed by first estimating the fragment size using cross-correlation analysis from the phantompeakqualtools software package (Landt et al., 2012). Peaks were called using MACS 2 on merged sample replicates against merged control replicates (Zhang et al., 2008). The following arguments were used: --g mm --keep-dup all -s < read length > --nomodel --shift 0 --extsize < fragment size > -p 0.05 -B --SPMR.

#### 2.9.1 Read coverage visualisation

DeepTools suite (Ramírez et al., 2016) was employed for read coverage visualisation. Normalized input-subtracted read coverage was calculated using merged control and samples with the ratio subtract and normalize to RPKM options. Heatmaps were generated using plotHeatmap command with the normalized input-subtracted read coverage and peaks called from the merged sample replicates. The DiffBind software package from the Bioconductor project (Ross-Innes et al., 2012) was used for correlation and principal component analyses of all ChIP-seq data.

#### 2.9.2 *De novo* motif analysis

Was performed with the findMotifsGenome tool of the HOMER (Heinz et al., 2010) suite searching for 250 bp around the peak summit. The background was generated by the HOMER software and only motifs with *p*-value < 1 × 10^−50^ were consider significant. The tool annotatePeaks.pl was also used to find specific motifs 250 bp around the peak summit.

#### 2.9.3 Overlapping peaks and peak to gene assignment

Peaks were considered to be overlapping when there was at least 1 bp of overlap between a 300 bp region centered on each peak summit. These overlapped peaks were obtained using mergePeaks tools from the HOMER software. Overlapping and unique peaks were annotated to the nearest TSS using ChIPseeker (Yu et al., 2015) package in Bioconductor. For most of the analyses, only peaks associated with differentially expressed genes were used for functional analyses that were carry out with clusterProfiler package (Yu et al., 2012) and topGO in Bioconductor (Alexa and Rahnenfuhrer 2019). Venn diagrams and histograms were generated using the package Vennerable (Swinton 2009) and ggplot2 in the R statistical environment.

### 2.10 GST pulldown assay

The GST-Gfi1 bacterial expression construct comprising the mouse Gfi1 coding sequence cloned in pGEX-KG between Bam HI and EcoRI sites was a kind gift from Dr Angela Chen and Dr Pin Yao Wang, Taiwan. The pGBKT7-myc-Atoh1 construct was made by ligating PCR amplified mouse Atoh1 coding sequence (primers in Supplementary Table S1) in pGBKT7 between EcoRI and NdeI. The GST Pulldown assay was carried out essentially as described in (Nguyen and Goodrich 2006) except lysis and wash buffers contained 5 mM β-mercaptoethanol rather than 1 mM DTT, and also contained 50 μM ZnSO_4_. Briefly, *E. coli* BL21 plysS cells were transformed with GST or GST-mGfi expression construct (pGEX-KG-GST or pGEX-KG-mGfi1 respectively) and grown overnight in L broth at 25°C. Protein expression was induced using 1 mM IPTG for 5 h at 25°C in the presence of 100 μM ZnSO_4_ before harvesting of the GST, or GST-Gfi1 containing bacterial pellet. The GST alone or GST-Gfi1 was bound to glutathione sepharose and treated with micrococcal nuclease to remove excess nucleic acid. Next a micrococcal nuclease-treated extract containing myc tagged Atoh1 produced from pGBKT7-myc-Atoh1 construct using the TNT Quick Coupled Transcription/Translation system (Promega) was incubated with the GST or GST-Gfi1 complexed to the GSH-sepharose beads for 2 h at 4°C. This was followed by extensive washing according to (Nguyen and Goodrich 2006), then the beads were boiled in SDS PAGE sample buffer and the resulting samples run on an SDS PAGE gel, subjected to Western blotting and the blot was probed with anti-myc antibody. A negative control pulldown was carried out in parallel with the unrelated ciliogenesis protein LRRC6 (results not shown).

### 2.11 Luciferase reporter assay

Mouse Gfi1 expression constructs used in P19 cells included pEx-Mm02706-M12 (Genecopoeia) and pCMV5-Gfi1 which was made by PCR amplification of the Gfi1 coding sequence and cloning in pCMV5 between MluI and XbaI (see Supplementary Table S1 for primers). pCMV-myc-Atoh1 was made by amplification of the Atoh1 coding sequence and cloning in pCMV-myc-N (Clontech) between EcoRI and XhoI. The Ascl1 expression construct pCDNA Mash1-HA, the hE47 expression construct pRC-CMV-hE47, and the Ascl1-specific E-box luciferase reporter construct pGL3-6*AsclE1 were kind donations from Castro et al. (2006). E-box concatemers used in luciferase reporter constructs were made for the following as described in Powell et al. (2004). pGL4.23-6*AtEAM: Atoh1 E-box Associated Motif according to (Klisch et al., 2011), concatemerized and cloned into pGL4.23 luciferase (Promega); R21 Gfi1 binding site (pGL3-6*R21), concatemerized and cloned into pGL3-promoter luciferase (Promega).

For P19 cells, Thermo Scientific Lipofectamine 3000 Reagent Kit was used for the transfection of 0.5 × 10^5^ cells with 550 ng total DNA in 24 well plates. The luciferase assay was conducted 48 h after the transfection (Promega Dual-Luciferase Reporter Assay System Kit). The ratio of firefly and Renilla fluorescence intensity was analyzed by GraphPad Prism, calculating the mean value and standard deviation from the three technical repeats in the same group. The mean value of 0 protein group was chosen as the base level, and the fold change of the mean value and deviation from each group was calculated and plotted as a bar chart. To test the significance, the original data from each group went through one-way ANOVA followed by Bonferroni correction, together with Tukey’s range test as a parallel check, taking 95% confidence interval.

### 2.12 Statistics

All qPCR data are expressed as mean ± s.e.m. and statistical significance was assessed using an unpaired Student’s *t*-test. For all statistics, data from at least three biologically independent experiments were used. Data and graphs were tabulated and prepared using Microsoft Excel and GraphPad Prism software. *p* < 0.05 was considered statistically significant.

## 3 Results

### 3.1 Gfi1 and Pou4f3 facilitate hair cell gene activation without repressing Atoh1’s neuronal programme

To investigate how Gfi1 and Pou4f3 modulate the lineage determining activity of Atoh1, we engineered mouse embryonic stem cell (ESC) lines that allow for dox-inducible co-expression of Gfi1, Pou4f3 and Atoh1 (iGPA) or Atoh1 alone (iAtoh1). In each polycistronic cassette, the transgenes were separated by the self-cleaving peptide, 2AP, and end with an mVenus fluorescent reporter (Figure 1A). To facilitate immunoprecipitation for subsequent ChIP-seq analysis, FLAG and MYC tags were appended to the C-terminus of Atoh1 and N-terminus of Pou4f3 respectively (Figure 1A). This double-tagged iGPA line was able to induce iHC differentiation with similar efficiency to that of the previously described untagged iGPA counterpart (iGPA-Myo7a:mVenus line (Costa et al., 2015) (Supplementary Figure S1). We also attempted to tag Gfi1 with an HA epitope, resulting in a triple-tagged iGPA cell line, but this line did not support iHC differentiation upon transgene induction, and therefore the double-tagged iGPA line was used in subsequent experiments (Supplementary Figure S1A).

To induce differentiation, embryoid bodies (EBs) were generated from iGPA and iAtoh1 lines and were then treated with Dox for 2, 4, and 8 days (Figure 1B). Gene expression analysis of mVenus^+^ cells revealed that while iGPA lines induced the expression of HC markers, iAtoh1 cells predominantly activated general neuronal genes (Figures 1C,D). This confirms that forced expression of Atoh1 alone promotes neuronal differentiation in agreement with previous studies using *in vitro* ESC differentiation assays (Srivastava et al., 2013; Sagal et al., 2014; Ebeid et al., 2017).

We set out to test the initial hypothesis that Gfi1 and/or Pou4f3 repress Atoh1’s neuronal programme during iHC differentiation. We performed RNA-seq in FACS-purified mVenus^+^ cells harvested 48 h after Dox induction and identified genes differentially expressed (DE) compared to non-dox treated EBs. As expected, DE genes in iGPA cells display a HC signature, while those in iAtoh1 cells showed a neuronal signature (Supplementary Figures S2A,B).

Unsupervised clustering of all DE genes (2,818 genes) revealed four prominent clusters (Figure 1E). Cluster 1 and 2 contained genes that are enriched in Dox-treated EBs relative to non-Dox-treated controls and these are mainly associated with neuronal and HC identity. On the other hand, genes within cluster 3 and 4 were mainly depleted relative to non-Dox controls and are not specifically associated with neurons or HCs. We focused our subsequent analysis on genes within cluster 1 and 2. Cluster 2 contains genes that were exclusively enriched in iGPA cells and are mainly associated with HC development, ion transport and sensory neuronal development (Figure 1E). Cluster 1 contains genes associated with neuronal development, which as expected were enriched in iAtoh1 cells (Figure 1E). Surprisingly, most of these genes (cluster 1A: 363 out of 511 genes) were also active in iGPA cells despite these cells not displaying an apparent neuronal identity. Only a small subset of neuronal genes including Barhl2, Tubb3, and Dcx were specifically activated in iAtoh1 cells and not in iGPA cells (cluster 1B; Supplementary Figure S2C). These findings indicate that iGPA cells differentiate to the HC lineage while retaining a large part of the neuronal program that is induced in iAtoh1 cells.

To determine whether this Atoh1 neuronal programme is also active during HC differentiation *in vivo*, we performed gene set enrichment analyses of clusters 1A, 1B and 2 on RNA-seq data previously obtained from HCs of the mouse inner ear (Scheffer et al., 2015). This revealed greater and more significant enrichments of clusters 1A and 2 genes compared to cluster 1B genes in both embryonic and postnatal HC transcriptomes (Figure 1F). The fact that genes in clusters 1A and 2 are equally enriched in native HCs at early stages of differentiation (HCs E16, Figure 1F) suggests that the dual activation of both neuronal and sensory differentiation programs is a feature of HC development *in vivo*. Conversely, the subset of Atoh1 neuronal targets that are repressed in *vitro* iHCs (cluster 1B) are also less abundant during early HC differentiation stages *in vivo*. Taken together, these data indicate that genes activated in response to Atoh1 include one set activated in both neuronal and HC contexts (cluster 1A) and another set activated in only the HC context (cluster 2). In addition, a smaller subset of genes activated by Atoh1 are repressed in the HC context (cluster 1B).

We conclude that, contrary to our initial hypothesis, the modulation of Atoh1 by Gfi1/Pou4f3 entails activation of a new HC gene expression programme but without concomitant widespread repression of Atoh1’s neuronal programme.

### 3.2 Gfi1 is critical for the switch from neuronal to hair cell fate

**FIGURE 2:**
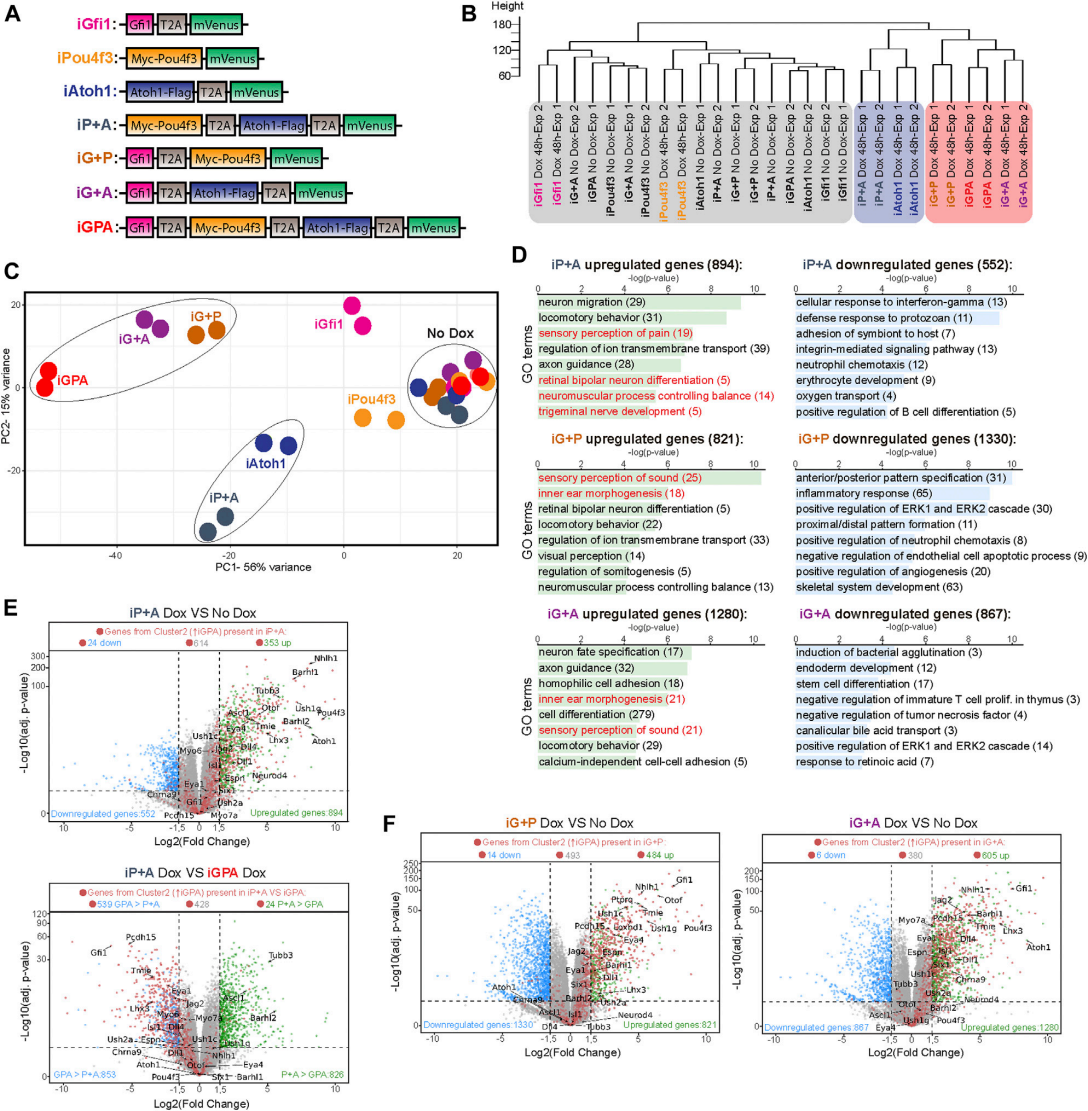
Transcriptome analysis of differentiation supported by TF combinations reveals Gfi1 is critical for HC switch. **(A)** Diagram of the Dox-inducible tagged TF constructs used to engineer the seven different ESCs lines capable of overexpressing different TF combinations upon Dox treatment. T2A, 2A self-cleaving peptide derived from thosea asigna virus. **(B)** Dendrogram showing hierarchical clustering of the various expression profiles obtained from EBs induced to express different TF combinations, as indicated in **(A)**. 48 h of Dox-treated EBs and no Dox exposed EBs were collected at day 6 of culture for transcriptome analysis. Exp1 and Exp2 corresponds to two biological replicates. **(C)** Principal component analysis (PCA) obtained from the transcriptome profiles of all Dox-inducible EBs lines that contained different TF combinations. EBs were grown in the presence or absence of Dox for 48 h before collection at day 6 of culture. The PCA explain 56% (PC1) and 15% (PC2) of total variance. **(D)** Gene ontology (GO) analysis performed for all the differentially expressed genes of each cell line iP+A, iG+P and iG+A when comparing Dox treated EBs versus non-treated EBs at day 6. The total number of selected differentially expressed genes (adjusted *p*-value ≤ 0.05 and log2-fold change ≤ −1.5 or ≥ 1.5) are indicated. **(E)** Volcano plots showing the log2-fold change of mRNA transcript levels and the log10-adj. *p*-value obtained when comparing iP+A-Dox treated EBs versus iP+A control EBs (not treated) as well as comparing iP+A-Dox treated EBs versus IGPA Dox treated EBs at day 6 after 48 h of Dox exposure. Each dot represents an expressed gene. Blue and green dots indicate genes significantly downregulated (log2-fold change ≤ −1.5 and *p*-value ≤ 0.05) and upregulated genes (log2-fold change ≥ 1.5 and *p*-value ≤ 0.05), respectively. Red dots represent the genes previously identified in cluster 2 (C-2: containing genes enriched only in GPA from Figure 1E). Total counts of the red dots for each category (downregulated, upregulated and non-differentially expressed genes) are show on top of each volcano plot. **(F)** Volcano plot shown as described in **(E)** but highlighting the differentially expressed genes in Dox treated iG+P EBs compared with no Dox control iG+P EBs as well as Dox treated iG+A EBs compared with no Dox control iG+A EBs.

To investigate the individual contributions of Pou4f3 and Gfi1 to modulating Atoh1-induced differentiation, we generated cell lines that allow inducible expression of every combination of the three TFs (Figure 2A). We compared global transcriptional changes using hierarchical clustering and principle component analysis. On their own, Gfi1 or Pou4f3 possess little capacity to modify gene expression (iGfi1 and iPou4f3 in Figures 2B,C). Surprisingly the other TF combinations resulted in gene expression programmes that clustered into two distinct groups correlating with the presence or absence of Gfi1 (Figures 2B,C).

The transcriptome of iP+A cells resembles that of iAtoh1 cells rather than that of iGPA cells, suggesting a shared neuronal identity (Figures 2B,C). Consistent with this, Atoh1 + Pou4f3 were unable to induce HC markers in EBs even after 8 days of Dox treatment. Rather, general neuronal markers were upregulated in iP+A cells even more strongly than in iAtoh1 cells (Supplementary Figure S3A). Therefore, Pou4f3 alone cannot convert Atoh1 from a neuronal determinant to a HC determinant.

Pou4f3 alone is, however, able to modify Atoh1’s neuronal programme. Most strikingly, the genes enriched in iP+A but not iAtoh1 are involved in the development of sensory neurons (Figure 2D) and are associated with sensory functions such as “perception of pain” (Supplementary Figure S3B). Although iP+A did not induce HC differentiation, we were able to detect 35% of genes from the HC-related DE gene cluster 2 amongst the genes upregulated by iP+A (Figure 2E). However, their expression remained considerably lower than in iGPA cells (Figure 2E) and seemed to be insufficient to trigger the activation of the HC differentiation program. Altogether, these data suggest that the co-expression of Atoh1 and Pou4f3 promotes the activation of a sensory neuronal program, not a functional HC programme.

In contrast, expression profiles of iG+P and iG+A cells cluster strongly with iGPA cells, suggesting that Gfi1 in combination with either Atoh1 or Pou4f3 or both drives cells to a common differentiation path related to HCs (Figures 2B,C). The presence of Gfi1 therefore contributes to a major shift in gene expression when it is combined with Atoh1 and/or Pou4f3 (Figure 2C). This differentiation pathway is characterised by activation of genes required for sensory perception of sound, consistent with HC identity (Figures 2D,F). These observations support a model in which Gfi1 dramatically modulates the transcriptional activity of both Atoh1 and Pou4f3 to enable HC differentiation.

**FIGURE 3:**
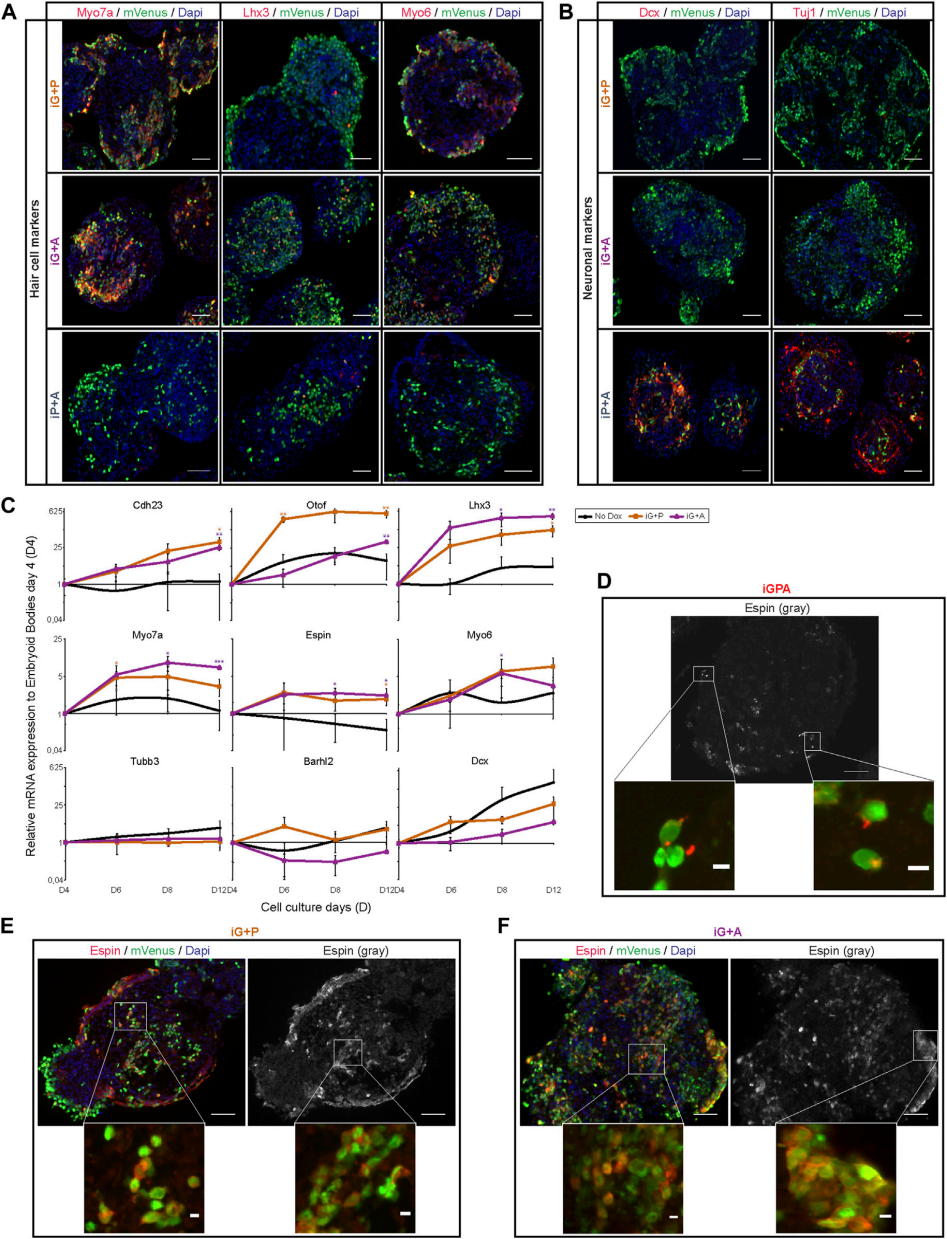
Gfi1 with Atoh1 or Pouf43 promotes HC gene expression but cellular differentiation requires all three TFs. **(A)** Representative images obtained from immunostaining of HC markers (Myo7a, Lhx3, and Myo6) (red) in induced EBs at day 8 of culture after 4 days of Dox treatment. mVenus (green) indicates cells overexpressing the transgenic TF combination and nucleus are labelled by DAPI (blue). Scale bar, 50 µm. **(B)** Expression of neuronal markers (Dcx and Tuj1) (red) in the same induced EBs shown in **(A)**. Scale bar, 50 µm. **(C)** qRT-PCR analyses for HC markers (Cdh23, Otof, Lhx3, Myo7a, Espin, and Myo6) and neuronal markers (Tubb3, Barhl2, and Dcx) in EBs derived from iG+P and iG+A ESC lines after 2, 4, and 8 days of Dox treatment (2 ug/ml of Dox was added at day 4 of EB culture and analyses were carried out at day 6, 8, and 12 of EB culture, respectively). Relative expression of each transcript is presented as fold change normalized to the mean of EBs at culture day 4 before Dox treatments. Results are mean ± s.e.m. *p < 0.1, **p < 0.01, ***p < 0.001 (*n* = 3, biological replicates). **(D)** Espin expression in polarised stereocilia-like outgrowths in iGPA cells at day 12 (EBs exposed to 8 days of Dox treatment). Scale bar: 50 µm for gray Espin picture and 5 µm in red Espin staining in the mVenus + cells. **(E,F)** Espin expression is not polarised in iG+P **(E)** or iG+A **(F)** EBs at 12 days of culture and 8 days of Dox exposure. Scale bar: 50 µm and 5 µm for the magnified square pictures.

The ability of Gfi1 + Pou4f3 to drive expression of HC genes in the absence of exogenous Atoh1 was a surprise. We therefore examined the efficiency of HC differentiation in iG+P cells using immunostaining and qPCR for HC and neuronal markers. This confirmed that Gfi1 abolishes the neurogenic activities of both Atoh1 and Pou4f3 and enables each of them instead to initiate an HC differentiation programme (Figures 3A–C). However, we found that HC differentiation was not as efficient in either iG+P or iG+A cells as it was in iGPA cells (Figure 1C). For example, the HC marker Lhx3 was confined to a minor subset of mVenus^+^ cells in both iG+P and iG+A lines (Figure 3A). Furthermore, iG+P and iG+A cells were unable to develop Espin^+^ hair bundle-like structures, in contrast to iGPA cells which display a clear polarised Espin signal (Figures 3D–F; Supplementary Figure S1A). Instead, Espin staining was spread throughout the cytoplasm of the mVenus^+^ iG+P and iG+A cells. This suggests that despite the expression of hair bundle genes (Figure 3C), key components necessary for organization of these hair-bundle-like protrusions are likely to missing from iG+P and iG+A cells.

In summary, we find that complete activation of the iHC differentiation programme requires all three TFs. Gfi1 plays a critical role by imposing a dramatic change on the transcriptional activity of both Atoh1 and Pou4f3, thereby allowing these TFs to activate a partial HC differentiation program individually, or a more complete HC programme together.

### 3.3 Atoh1 directly regulates both neuronal and sensory genes

**FIGURE 4:**
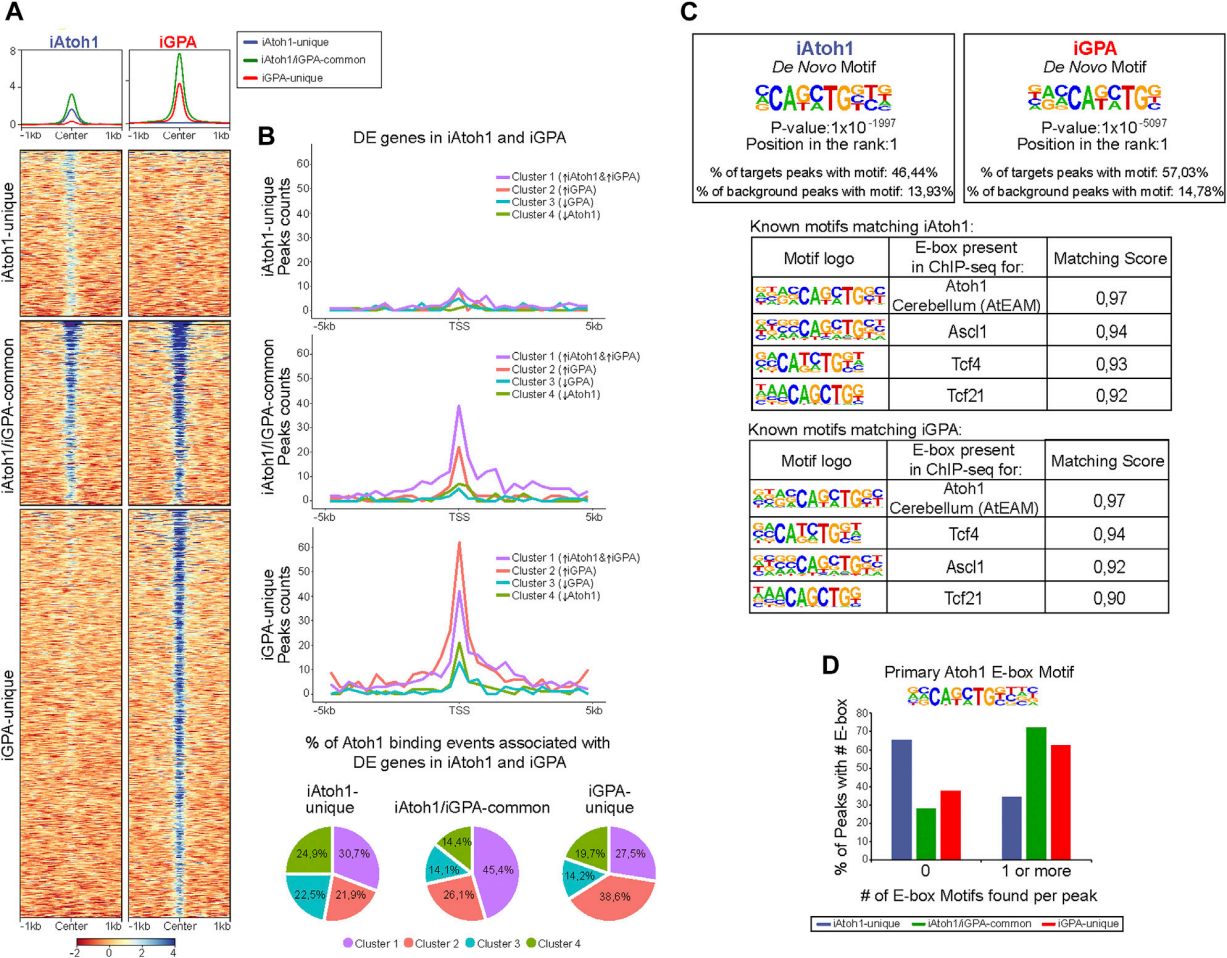
Gfi1+Pou4f3 promote Atoh1 binding to its preferred motif at new genomic locations without inhibiting binding to its original targets. **(A)** Density heatmaps of Atoh1 Chip-seq datasets from iAtoh1 and iGPA EBs at 48 h post-induction of TF overexpression. Each column shows the Atoh1 ChiP-seq signal for iAtoh1 and iGPA lines which are divided by the three different Atoh1 peak groups identified in (Supplementary Figure S4A). Each row represents the normalized counts (reads per kilobase per million, RPKM) for Atoh1 ChIP-seq peak signal spanning ± 1 kb from the centre of the peak. **(B)** Plot representing the total peaks counts found ± 5 Kb around the transcription start site (TSS) for the previously identified gene clusters in iAtoh1 and iGPA RNA-seq datasets (Figure 1E). Peak counts were divided into three groups (iAtoh1-unique, iAtoh1/iGPA-common and iGPA-unique) shown in three separated plots. The number of peaks found for each gene cluster was divided by the total number of peaks of each category to obtain the % Atoh1 binding associated with DE genes of iAtoh1 and iGPA EBs represented in pie charts. **(C)** Motifs analyses for Atoh1 peaks in iAtoh1 and iGPA EBs. Upper: the most significant *de novo* motif detected in iAtoh1 and iGPA by searching at 250 bp regions centred at summits; lower: best match between known motifs and motifs found in Atoh1 peaks shows that the previously detected Atoh1 motif in cerebellum (AtEAM) has the highest match score in both iAtoh1 and iGPA EBs. **(D)** Graph showing the number of AtEAM motifs found in each Atoh1 peak group: iAtoh1-unique, iAtoh1/iGPA-common and iGPA-unique.

Having identified genes upregulated in response to Atoh1 in the contexts of neuronal or HC differentiation, we asked which of these genes might be directly regulated by Atoh1. Taking advantage of the FLAG-tagged Atoh1, we carried out Atoh1 ChIP-Seq on both iAtoh1 and iGPA lines after inducing transgene expression in EBs for 48 h. 15,568 genomic sites were enriched for Atoh1 in iAtoh1 cells and 26,231 sites in iGPA cells. Together, these sites could be divided into three groups (Supplementary Figure S4A): iAtoh1-unique (7,279 sites), iAtoh1/iGPA-common (8,056 sites), and iGPA-unique (17,851 sites) (Figure 4A). We examined whether these differences in Atoh1 binding explain how Atoh1 switches from neuronal to HC determinant.

To determine whether these groups are associated with the DE gene clusters identified above, we integrated Atoh1 genomic location analyses with these DE gene clusters by annotating the peaks to their closest TSS. First, the iAtoh1-unique group of sites showed relatively low binding enrichments and were not preferentially associated with any particular DE gene cluster (Figures 4A,B). We therefore surmise that this group likely reflects weak and non-functional binding of Atoh1. We next examined iAtoh1/iGPA-common binding sites. These were predominantly associated with DE cluster 1 (enriched in neuronal genes), indicating that Atoh1 directly binds and activates neuronal genes regardless of the presence or absence of Pou4f3 and Gfi1. Consistent with this, iAtoh1/iGPA-common sites included enhancers that were previously shown to be enriched for Atoh1 in the developing cerebellum (Klisch et al., 2011) intestinal crypts (Kim et al., 2014) and spinal cord (Lai et al., 2011) (Supplementary Figure S4B), validating our ChIP-seq strategy using the FLAG tag. Finally, the iGPA-unique sites were predominantly associated with genes of DE cluster 2 (enriched in HC genes) (Figure 4B), suggesting that Atoh1 directly binds and activates HC genes but only in the presence of Pou4f3/Gfi1. It is not possible to determine from these data whether it is Pou4f3 or Gfi1 or both that enable Atoh1 to bind HC genes.

In *de novo* motif analysis of Atoh1 binding peaks, the best hit was an E box motif that matched most closely the AtEAM variant previously identified from Atoh1 binding sites in the cerebellum^6^ (Figure 4C; Supplementary Figure S5). More than 60% of iAtoh1/iGPA-common and iGPA-unique peaks contained one or more AtEAM E-box motifs compared to just 34.5% of iAtoh1-unique peaks (Figure 4D). This is consistent with the previous conclusion that the majority (65.5%) of iAtoh1 unique peaks largely represent low affinity and non-specific interactions. Altogether, these data suggest that Pou4f3 and/or Gfi1 do not interfere with Atoh1 binding at neuronal targets with its preferred E-box motif, but enable Atoh1 to bind to HC targets containing its preferred E-box motif, while depleting Atoh1 from weakly-bound sites which for the most part lack the E-box motif.

### 3.4 Atoh1 recruitment to new targets is largely driven by Pou4f3, not Gfi1

**FIGURE 5:**
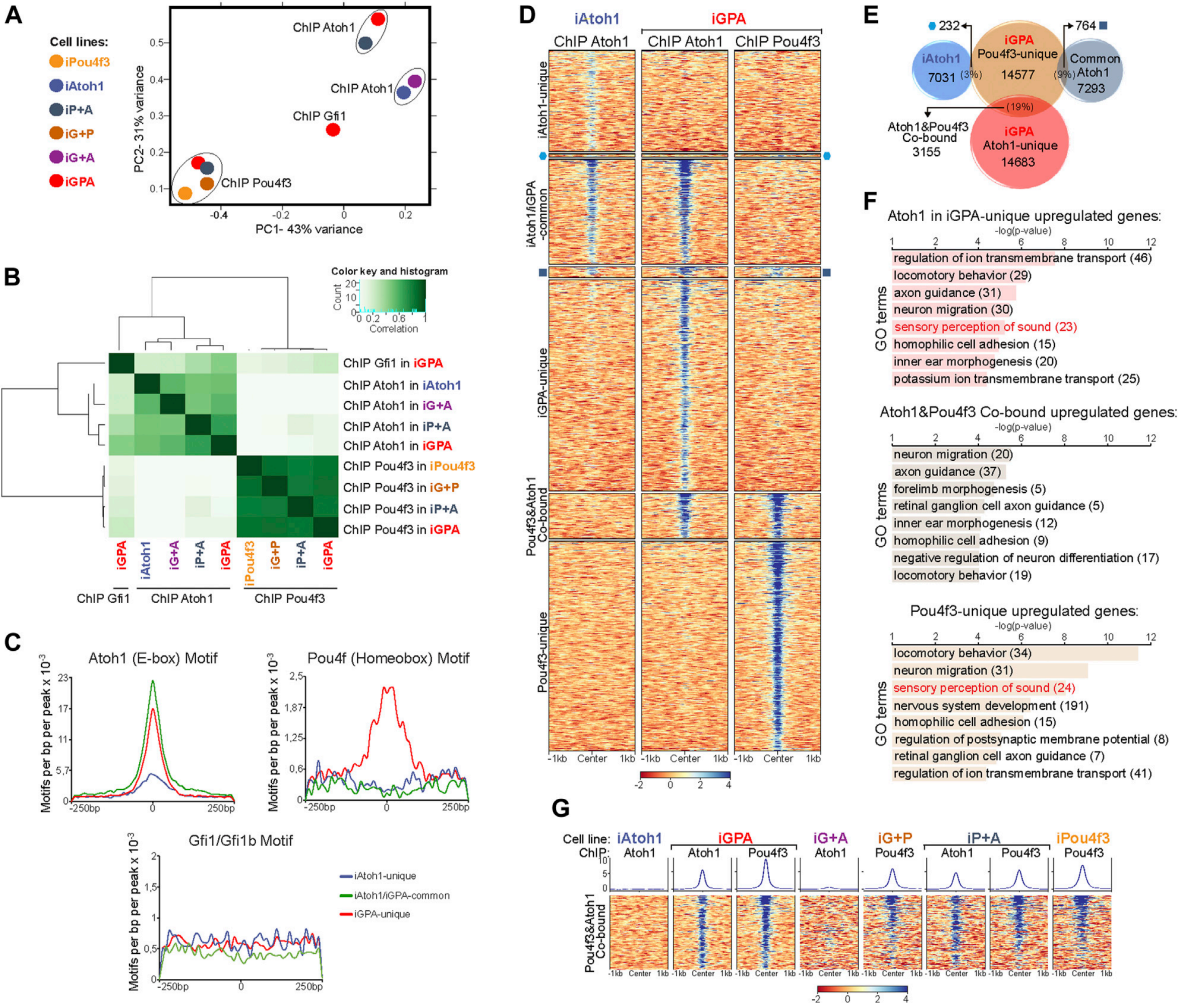
Pou4f3 recruits Atoh1 to new binding locations but not *vice versa*. **(A)** Principal component analysis of Atoh1, Pou4f3 and Gfi1 ChIP-seq peaks for each EB overexpressing combinations of the three TFs (48 h post-Dox treatment) as indicated by the colour key. Gfi1 ChIP-seq was only performed in iGPA EBs but ChIP-seq for Atoh1 and Pou4f3 was carry out in EBs overexpressing all 4 possible TF combinations. **(B)** Correlation heatmap of Atoh1, Pou4f3, and Gfi1 ChIP-seq peaks for each EB overexpressing combinations of the three TFs. **(C)** Frequency and distribution of Ebox, Pou4f homeobox and Gfi1 motifs across a 250 bp interval surrounding the Atoh1 peak centres for each Atoh1 peak group identified in (Figure 4A) **(D)** Density heatmaps of Atoh1 Chip-seq obtained from iAtoh1 and iGPA EBs and Pou4f3 ChIP-seq from iGPA EBs. The heatmap is divided in seven parts containing the overlapping and non-overlapping peaks across the 3 ChIP-seq datasets as represented in **(E)**. **(E)** Peak overlap between Pou4f3 ChIP-seq in iGPA EBs and Atoh1 ChIP-seq in both iAtoh1 and iGPA EBs. The number of unique and overlapping peaks across these 3 ChIP-seq datasets is indicated in the diagram and their ChIP signal is shown in **(D)**. **(F)** Gene ontology (GO) analysis performed for unique and common peaks of Atoh1/Pou4f3 ChIP-seq in iGPA EBs that were only associated with differentially expressed genes found in iGPA RNA-seq. Peaks were associated with genes following the nearest gene annotation rule. **(G)** Density heatmaps of Atoh1 and Pou4f3 ChIP-seq enrichment only at Atoh&1Pou4f3 cobound peaks (3,155 genomic regions) in different induced EBs. Peak signal (in colour scales) represents the normalized counts (reads per kilobase per million, RPKM) and is spanning ± 1 kb from the centre of the peak.

We next determined the relative importance of Pou4f3 and Gfi1 in directing Atoh1 to new binding sites during iHC differentiation. We performed ChIP-seq for Atoh1, and also for Pou4f3, after induction of all combinations of the three TFs. PCA and hierarchical clustering revealed that global Atoh1 binding is distinctly altered by the presence of Pou4f3 (iP+A and iGPA binding cluster distinctly from iAtoh1 binding) (Figures 5A,B). In contrast, the presence of Gfi1 does not strongly affect the profile of Atoh1 binding, regardless of the presence or absence of Pou4f3 (Atoh1 profiles in iAtoh1 vs. iG+A and in iGPA vs. iP+A, Figures 5A,B). Therefore, despite Gfi1’s importance for promoting the HC differentiation programme, the presence of Gfi1 did not make a large difference to the binding pattern of Atoh1.

In contrast to Atoh1, Pou4f3 binding profiles in all conditions cluster tightly together, suggesting that Pou4f3 binding is not strongly affected by the presence of either Atoh1 or Gfi1 (Figures 5A,B). Taken together our data suggest that the binding of Atoh1 to new sites during HC differentiation depends directly or indirectly on Pou4f3 function to a large extent. In contrast, and contrary to expectation, Gfi1 allows Atoh1 and Pou4f3 to activate a new transcriptional programme largely without redirecting their binding to new loci.

### 3.5 Pou4f3 directly recruits Atoh1 to a subset of new neuronal targets

The genomic binding profile of Pou4f3 is quite different from Atoh1 (Figures 5A,B), suggesting that much of the effect of Pou4f3 on Atoh1 binding redistribution might be indirect. To investigate the possibility of direct interactions, we searched for the enrichment of motifs in addition to E boxes in each of the three different groups of Atoh1 peaks. A homeobox motif bound by Pou4f TFs was the most significant secondary motif found in iGPA-unique peaks but was absent from the other peak categories (Figure 5C; Supplementary Figure S5B). This is consistent with the possibility that Pou4f3 might directly promote Atoh1 binding to new regulatory elements. To explore this, we performed clustering of the genomic locations for Atoh1 and Pou4f3 in the different TF combinations (Figures 5D,E). This reveals that Atoh1 co-binds with Pou4f3 at a subset of sites (3155 sites in cluster_3). The majority of these iGPA co-bound sites contained both Pou4f and E-boxes motifs with similarly high abundance (Supplementary Figures S6A,B), suggesting that co-binding of Atoh1 and Pou4f3 is dependent on the presence of both their specific motifs in the same regulatory element.

Notably, these co-bound sites are bound by Pou4f3 in all conditions (and therefore are not dependent on the presence of Atoh1), whereas binding of Atoh1 is prominent in iGPA and iP+A but not iAtoh1 or iG+A cells (Figure 5G). We conclude that Atoh1 binding is dependent on Pou4f3 but not *vice versa*, consistent with the idea that Pou4f3 recruits Atoh1 to these new sites.

To explore whether the cluster of Atoh1+Pou4f3 co-bound sites includes genes activated upon HC differentiation, we integrated the RNA-seq and ChIP-seq datasets. We annotated the peaks in each category and performed GO analyses only for genes previously identified as DE in iGPA cells. This analysis revealed that DE genes with GO terms related to HC differentiation were mostly bound independently by Atoh1 or Pou4f3 (unique sites) with only a minor fraction of HC genes co-bound by both TFs (Figure 5F). Instead GO terms related to neuronal migration and axon guidance were most significant among the co-bound sites (Figure 5F). Overall, these data suggest that Pou4f3 directly recruits Atoh1 to new loci, but these are associated with neuronal rather than HC differentiation. In contrast, recruitment of Atoh1 to HC loci by Pou4f3 therefore appears to be largely indirect.

### 3.6 Gfi1 co-binds with Atoh1 at a subset of sites

Having shown that Gfi1 is critical for converting Atoh + Pou4f3 from neuronal determinants to HC determinants, it was surprising that it does not appear to redistribute Atoh1 or Pou4f3 binding. Moreover, Atoh1 binding peaks in iGPA cells are not associated with Gfi1 motifs (Figure 5C). We therefore considered a model in which Gfi1 functions in parallel to and independently of Atoh1/Pou4f3. One prediction of this model is that Gfi1 does not co-bind with Atoh1 or Pou4f3 during HC differentiation. To test this, we performed ChIP-seq for Gfi1 in iHCs generated from the iGPA line. Since we were unable to epitope tag Gfi1, we relied instead on a Gfi1 antibody for immunoprecipitation. This antibody gave a relatively low binding enrichment over the input DNA, resulting in only 3003 detectable peaks. Nevertheless, Gfi1-unique peaks (those that did not overlap with Atoh1 or Pou4f3) were strongly enriched in Gfi1 DNA motifs (see below), pointing to the specificity of binding.

**FIGURE 6:**
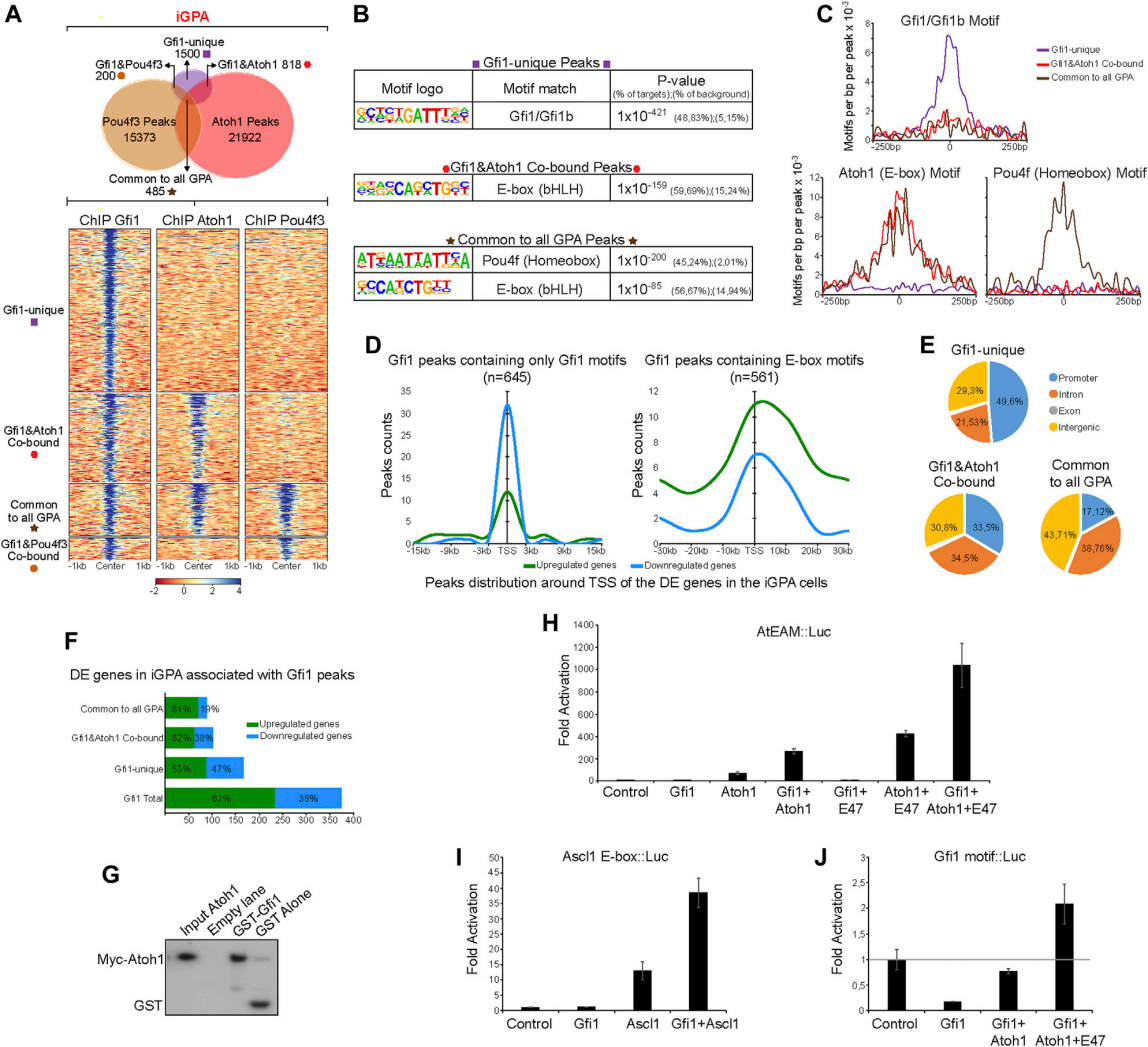
Gfi1 unique sites are enriched in Gfi1 motifs but Gfi1/Atoh1 cobound sites are enriched in E boxes. **(A)** Upper: Venn diagram showing the peaks overlap between Gfi1, Pou4f3, and Atoh1 ChIP-seq in iGPA EBs. Lower: Density heatmaps of Gfi1, Atoh1, and Pou4f3 ChIP-seq obtained from iGPA EBs 48 h post-induction of TF overexpression. The heatmap is divided by non-overlapping and overlapping peaks across these 3 ChIP-seq datasets as represented in the upper Venn diagram. **(B)** *de novo* motifs analyses showing the most significant motifs found in Gfi1 ChIP-seq dataset which was divided by Gfi1-unique peaks, Gfi1&Atoh1 co-bound and Gfi1&Atoh1&Pou4f3 co-bound (common to all GPA) peaks. **(C)** Frequency and distribution of Gfi1, Ebox, and Pou4f homeobox motifs across a 250 bp interval surrounding Gfi1 peak centres for each Gfi1 unique and overlapping peak groups. Gfi1 mofifs are highly enriched in the Gfi1-unique peaks but not in Gfi1 overlapping peaks where Ebox and homeobox motifs are enriched. **(D)** Gfi1 peaks divided by either containing Gfi1 motifs or Ebox motifs were associated with gene repression (blue) or activation (green) among the DE genes found in iGPA EBs. Smooth curves show higher numbers of peaks containing Gfi1 motifs associated with gene repression only close to TSS. In contrasts, the majority of Gfi1 peaks containing Ebox are associated with gene activation both close and outside (+/30 kb) of TSS. **(E)** Pie charts showing the location of Gfi1 unique and overlapping peak relative to several genomic features. **(F)** Percentages and numbers of downregulated and upregulated genes among the DE genes in iGPA EBs associated with Gfi1 peaks detected in iGPA cells. **(G)** GST pull-down analysis of Myc-tagged Atoh1 with GST-tagged Gfi1. Western blot probed with anti-Myc antibody. **(H)** Expression analysis of luciferase reporter gene under control of multimerised Atoh1 (AtEAM) motifs in cells cotransfected with expression vectors for Gfi1, Atoh1 and/or E47 (control is cotransfection with empty expression vector). Fold activation shown is relative to control. The data with E47 are from a separate experiment from the data without E47. **(I)** Similar to **(H)** but with a luciferase reporter gene under control of multimerised Ascl1 motifs. **(J)** Similar to **(H)** but with a luciferase reporter gene under control of multimerised Gfi1 binding motifs.

We analysed these Gfi1 binding peaks for overlap with the other two TFs. To our surprise, 43% of these peaks overlapped with those of Atoh1 Figure 6A. Furthermore, Pou4f3 peaks overlapped with Gfi1 peaks mostly only where Atoh1 peaks were also present. Motif analysis revealed that Atoh1-Gfi1 co-bound peaks are not enriched in Gfi1 DNA motifs (Figures 6B,C). This corroborates our previous finding that Atoh1 and Pou4f3 peaks lack Gfi1 binding motifs (Supplementary Figure S6A). Instead, E-boxes featured prominently in those Gfi1 peaks that coincide with Atoh1 peaks. Pou4f motifs were also detectable, but only in peaks in common to all three TFs (Figures 6B,C). Together, these data suggest that Gfi1 is recruited to a set of loci through interaction with Atoh1 rather than through specific DNA binding. In support of this hypothesis we were able to recover Myc-tagged Atoh1 from an *in vitro* pulldown assay using a recombinant GST-Gfi1 protein (Figure 6G).

In addition to the overlapping Gfi1 peaks above, 50% of Gfi1 binding sites did not overlap with those of Atoh1 or Pou4f3. In contrast to the overlapping peaks, these “Gfi1-unique” sites were enriched in Gfi1 motifs (Figures 6B,C). Together, these findings raise the possibility that Gfi1 binds to one set of target sites directly and independently but is recruited to another set of loci through interaction with Atoh1.

### 3.7 Gfi1 regulates hair cell differentiation through both repression and Atoh1 co-activation

Gfi1 protein contains a SNAG domain which recruits co-repressors (Grimes et al., 1996; Zweidler-Mckay et al., 1996; Velinder et al., 2016), and it acts as a major transcriptional repressor during haematopoiesis (van der Meer et al., 2010; Möröy and Khandanpour 2011; Möröy et al., 2015). However, the *Drosophila* orthologue of Gfi1 (Senseless, SENS) (Nolo et al., 2000) has been reported to have dual functionality: SENS can act as a DNA-binding transcriptional repressor but also as a transcriptional co-activator of proneural TFs (including Atonal), enhancing their ability to stimulate sensory gene expression (Jafar-Nejad et al., 2003; Acar et al., 2006; Powell et al., 2008; Powell et al., 2012). Our observation of two distinct populations of Gfi1 sites in iHCs suggests that Gfi1 may similarly have dual functionality. We hypothesised that 1) Gfi1 represses genes that it binds to through its own motif independently of Atoh1, and 2) interaction between Gfi1 and Atoh1 at Atoh1-dependent sites switches Gfi1 from a repressor to a co-activator.

To test this hypothesis, we separated the Gfi1 peaks into two groups: one containing only Gfi1 motifs and the other containing E-box motifs, and assessed whether these were associated with genes that were activated or with genes that are repressed during iHC differentiation of iGPA cells. Consistent with Gfi1’s known role in hematopoiesis, peaks with Gfi1 motifs appeared to be strongly associated with gene repression, albeit only when located near to the TSS of the assigned genes (Figure 6D). In support of this, we observed that 49.6% of Gfi1-unique sites are located near promoters and around 47% of all Gfi1-unique sites are associated with downregulated genes (Figures 6E,F). In notable contrast, the majority of Gfi1 peaks with E-boxes (i.e., Gfi1-Atoh1 co-bound sites) are associated with up-regulated genes (Figure 6D). Interestingly, these Gfi1 and Atoh1 co-bound sites do not show a preference for promoters and are more frequently located in intergenic and intronic regions (Figure 6E). These findings support the hypothesis that Atoh1 can switch Gfi1 from a repressor to a co-activator.

We then looked for evidence for the different roles of Gfi1 by examining DE genes. We performed unsupervised clustering of all genes differentially expressed relative to no-dox controls in the iGfi1, iAtoh1, iG+A and iGPA lines. A total of 1,921 genes were clustered into five groups in accordance with their shared expression patterns across these four lines. Group 4 includes a set of Atoh1-responsive genes that are repressed by the presence of Gfi1 (Supplementary Figure S7A: Group 4). We were able to detect Gfi1 binding at the vicinity of some group 4-genes, including several Hox and Id genes, as well as TFs important for lineage differentiation (e.g., Otx1 and NeuroD4) (Supplementary Figure S7B). This supports the suggestion that part of Gfi1’s function is the direct repression of non-HC targets. In addition, groups 1 and 2 contain genes that require Gfi1 and Atoh1 for their upregulation. Interestingly, Gfi1 enhanced the expression of common Atoh1 target genes (Supplementary Figure S7A: Group 1-Notch and neuronal genes), as well as enabling expression of a group of genes that Atoh1 by itself is not able to activate (Supplementary Figure S7A: Group 2). Notably, this included genes associated with inner ear morphogenesis. However, the other HC-related GO term ‘sensory perception of sound’ is only associated with genes upregulated in the presence of all three TFs (Group 3), supporting the phenotypic observations that more complete iHC differentiation requires Pou4f3 in addition to Gfi1 and Atoh1.

To further test the hypothesis that Gfi1 is both a repressor and an Atoh1-dependent co-activator, we performed transcriptional assays in P19 cells transfected with reporter constructs bearing luciferase with a minimal promoter sequence under the regulation of multimerised binding sites for Gfi1 or Atoh1. We first tested the effect of Gfi1 expression on the expression of luciferase under control of a promoter that consisted of strong Gfi1 binding sites (R21). Consistent with the findings of previous studies, Gfi1 was able to repress expression of the R21 reporter gene efficiently (Grimes et al., 1996; Zweidler-Mckay et al., 1996) (Figure 6J). This corroborates our observation in iGPA cells that Gfi1 binding to its own motif is associated with transcriptional repression. We then tested a reporter gene under the control of multimerised Atoh1 E box binding sites (AtEAM)^6^. As expected, expression of Atoh1 on its own was able to induce AtEAM reporter gene expression and this effect was further enhanced by adding an E-protein (E-proteins are essential heterodimerisation partners of Atoh1) (Figure 6H). Expression of Gfi1 alone had no effect on reporter activity consistent with inability to bind the E box, but remarkably co-expression of Gfi1 and Atoh1 significantly augmented reporter gene expression. This reveals a synergy in activity between these two TFs (Figure 6H). Similar results were observed when Gfi1 was co-expressed with another proneural bHLH factor, Ascl1, suggesting that such synergistic effect could occur more broadly in other cell fate decisions requiring proneural TFs (Figure 6I). Interestingly, expression of Atoh1 is also able to counteract Gfi1 repression of the R21-regulated reporter gene, presumably because Atoh1 binds directly to Gfi1. Such a derepression effect might explain why we still detect 53% of genes up-regulated when Gfi1 is bound to their regulatory regions *via* its DNA motifs (Figure 6F) in the presence of Atoh1.

Taken together, these findings suggest that Gfi1 has a dual capacity to 1) act as a transcriptional repressor when bound to its DNA motifs located at promoter regions; 2) act as a transcriptional co-activator when interacting with Atoh1.

## 4 Discussion

**FIGURE 7:**
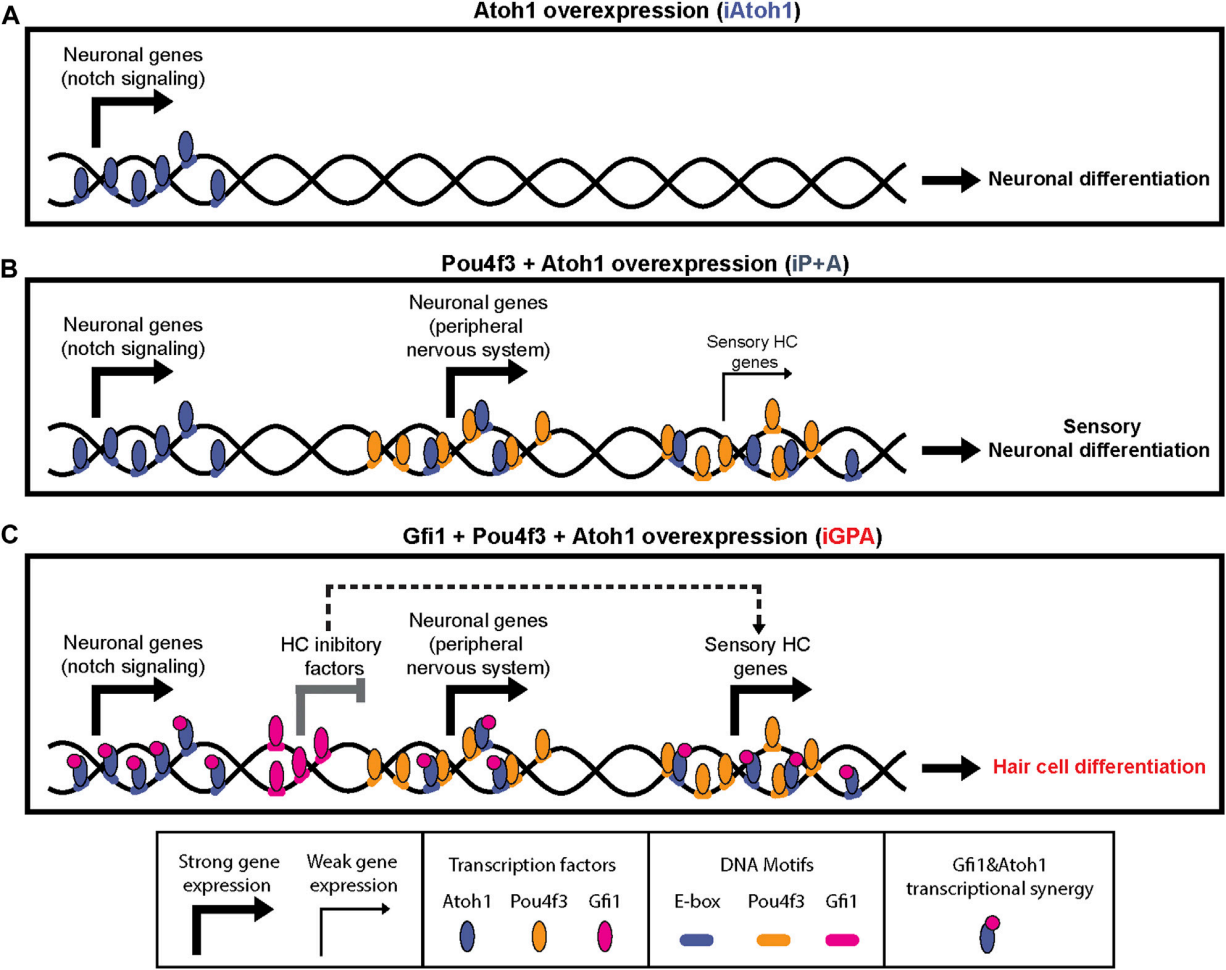
Pou4f3 and Gfi1 contribute different mechanisms in converting Atoh1 to an HC determinant in mESCs. Schematic summary of findings and possible interpretation. **(A–C)** Atoh1 binds to neuronal targets in all conditions. **(B)** Presence of Pou4f3 recruits Atoh1 (partly directly) to sensory targets (neuronal and HC) but this does not result in strong HC gene upregulation. **(C)** Gfi1 has a dual function in HC differentiation: it binds to and represses some targets that would otherwise interfere with HC differentiation; it cobinds *via* interaction with Atoh1 to sensory and HC targets where it promotes upregulation.

Although the lineage-determining ability of TFs is often modulated by working in combination with other TFs, the mechanisms by which such switching occurs are not well known. In this study using a model *in vitro* system, we found that Atoh1 is repurposed from a neuronal to HC determinant by the combined activity of Gfi1 and Pou4f3. Our evidence suggests that Atoh1 repurposing entails two major mechanisms: 1) allowing Atoh1 access to genomic locations controlling the expression of sensory genes; 2) enhancing Atoh1’s activity at its HC target genes. These two events are unlocked by Pou4f3 and Gfi1, respectively, and at least part of their effect is likely by direct interaction with Atoh1 (Figure 7).

### 4.1 Pou4f3 as a competence or pioneer factor for Atoh1

Pou4f3 expression enables the recruitment of Atoh1 to new genomic locations vital for HC differentiation. In the absence of Gfi1, however, this recruitment does not lead to full HC gene activation, rather to activation of sensory neuronal genes (Figure 7). Given the co-occurrence of their binding motifs, part of this recruitment (at co-bound sites) is potentially by direct interaction. It is particularly striking that Pou4f3’s binding activity is unaffected upon Atoh1 (or Gfi1) expression, and yet by itself it has little capacity for transcriptional regulation in ESCs (Figure 5C). Together, these observations suggest that Pou4f3 establishes lineage-specific competence for Atoh1-driven sensory cell differentiation, perhaps as a pioneer factor that allows Atoh1 access to its sensory targets. Consistent with this are reports that Pou domain TFs can reprogram mouse and human fibroblasts into iHC (Duran Alonso et al., 2018). A recent study of embryonic mouse cochlea cells corroborates our data by finding that about half of Atoh1 sites are inaccessible until Pou4f3 is activated (as a target of Atoh1 itself) (Yu et al., 2021).

Interestingly, this implies that Atoh1 is not a pioneer factor in HC differentiation *in vitro* (and perhaps *in vivo*). Indeed, *in vivo* evidence from intestinal crypt cell differentiation suggests that chromatin modification precedes Atoh1 binding (Kim et al., 2014). Together, this contrasts Atoh1 function with Ascl1, a related proneural factor that features heavily in “TF cocktails” for direct reprogramming of a variety of neuronal cell lineages (Masserdotti et al., 2016). Ascl1 appears to be a pioneer factor capable of binding nucleosome occluded DNA and of promoting chromatin accessibility at its targets in neuronal differentiation (Wapinski et al., 2013; Raposo et al., 2015).

Interestingly, whilst Pou4f3 recruits Atoh1 to new targets, it does not displace Atoh1 from its default neuronal targets. Lineage switching in iGPA cells therefore does not involve complete replacement of one programme by an alternative one. This may reflect the functional similarities between HCs and neurons, e.g., both require proteins for synaptic neurotransmission.

While a major effect of Pou4f3 may be to repurpose Atoh1 directly, it should be noted that many Pou4f3 sites are distinct from Atoh1 sites, suggesting that part of its function is indirect.

### 4.2 A dual function for Gfi1 as a transcriptional repressor and basic helix-loop-helix co-activator

In contrast to Pou4f3, Gfi1 does not alter Atoh1 binding sites. Instead Gfi1 appears to be recruited to Atoh1 sites where it acts as a co-activator of sensory genes. Thus overall, full HC determinant ability of Atoh1 requires Pou4f3 to guide it to new genomic sites, and Gfi1 to enable efficient activation of HC genes.

Our previous understanding of how Gfi1 regulates transcription largely comes from research into its role in hematopoietic development, where it acts as a major DNA-binding transcriptional repressor by recruiting chromatin regulatory complexes *via* its SNAG domain (McGhee et al., 2003; Duan et al., 2005; Saleque et al., 2007; Thambyrajah et al., 2016). Here we propose that Gfi1 additionally acts as a co-activator of Atoh1 target genes. The dual role of Gfi1 is supported in a study of mouse Gfi1 deficient HCs, which shows that it represses neuronal genes alongside activation of hair cell genes (Matern et al., 2020). Such a dual function for Gfi1 also has strong support from studies of the *Drosophila* orthologue, SENS (Nolo et al., 2000) during sensory neuron specification. As well as acting as a DNA-binding transcriptional repressor, SENS promotes the activity of bHLH proneural factors, including Atonal (Jafar-Nejad et al., 2003; Acar et al., 2006; Powell et al., 2008; Powell et al., 2012). In this role, SENS does not bind to its DNA motif, but directly binds proneural factors *via* its Zn-finger domains. Our findings suggest that such dual functionality of Gfi1/SENS is highly conserved during the specification of mechanosensory cells by Atoh1/Atonal. Indeed, a similar conclusion was reached in a recent study of Gfi1-deficient mice (Jen et al., 2022). Gfi1 differs from SENS in possessing a SNAG domain. This might suggest that binding to Atoh1 must mask the SNAG domain to prevent its repressive activity. Interestingly, Atonal binding to SENS also interferes with its ability to repress targets (Witt et al., 2010).

In *Drosophila*, interaction of SENS with proneural factors has been proposed to constitute a temporal switch from repressor to co-activator during sensory precursor specification (Jafar-Nejad et al., 2003). In our model, we suggest that Gfi1 repressor and co-activator roles are required concomitantly at different targets during HC differentiation. While co-activation is required for Atoh1-dependent HC gene expression, direct Gfi1-mediated gene repression is also likely to be important. A good example is the repression by Gfi1 of two bHLH TFs, Ascl1 and NeuroD4. These factors have the capacity to reprogram different cell types into neurons and so would be likely to favour Atoh1’s neurogenic activity unless repressed by Gfi1.

Future studies are needed to understand whether Gfi1 also acts as transcriptional co-activator in the hematopoietic and intestinal systems. Indeed, a few examples have been reported in which Gfi1 may activate gene expression during hematopoiesis, but in these cases Gfi1 appears to bind to its DNA motif (van der Meer et al., 2010). Atoh1/Gfi1 are co-expressed in several other cellular lineages, strongly suggesting that the synergistic mechanism may function beyond HCs. In cases where Gfi1 is not co-expressed with Atoh1 (e.g., hematopoiesis), it is possible that Gfi1 may similarly co-activate with other TFs.

### 4.3 Reprogramming by survival/maintenance factors: Implications for *in vitro* programming models and for hair cell gene therapy

*In vitro*, Gfi1 and Pou4f3 are crucial for repurposing Atoh1 for HC identity. In mouse knockout studies, however, both Gfi1 and Pou4f3 appear to be necessary for HC survival and differentiation rather than initial fate determination (Xiang et al., 1998; Wallis et al., 2003). It is possible that the embryonic otic environment has factors and mechanisms that can compensate for these functions of Pou4f3 and Gfi1. Moreover, for Gfi1, its Atoh1-synergising role may reflect their interaction at a later stage of lineage determination/maintenance *in vivo*. It is interesting to note that this parallels SENS function in *Drosophila*: SENS appears to function during sensory lineage determination by proneural factors (Jafar-Nejad et al., 2003; Acar et al., 2006; Powell et al., 2008) and yet SENS mutants mainly show defects in sensory lineage survival and differentiation (Nolo et al., 2000).

In this regard, the similarities between the iGPA strategy and other “reprogramming cocktails” is striking (Srivastava et al., 2013; Masserdotti et al., 2016). For instance, the original “ABM” regime for neuronal differentiation likewise consists of a bHLH proneural factor (Ascl1), a Pou domain TF (Brn2/Pou3f2) and Zn-finger TF (Myt1l) (Vierbuchen et al., 2010). Myt1l is suggested to safeguard neuronal identity during reprogramming by repressing non-neuronal lineages (Mall et al., 2017). Gfi1 may be acting similarly to repress inappropriate gene expression during HC differentiation.

The mechanisms by which Gfi1, Pou4f3 and Atoh1 orchestrate a reprogramming event towards HC fate may have implications for developing new therapies. HC loss is a major cause of human sensorineural hearing loss, which is permanent because HCs do not regenerate in mammals. There is much interest in inducing HC regeneration using vector-supplied *Atoh1* (Richardson and Atkinson 2015; Zhang et al., 2018). Rodent models demonstrate that cochlear overexpression of Atoh1 is sufficient to generate new ectopic HCs during embryonic inner ear development, but it fails at later postnatal/adult stages (Liu et al., 2012). Our findings that Pou4f3 facilitates Atoh1 binding to HC genes and Gfi1 efficiently enhances Atoh1 activation of HC genes suggest several avenues for enhancing the therapeutic potential of Atoh1.

## Data Availability

The datasets presented in this study can be found in online repositories. The names of the repository/repositories and accession number(s) can be found below: https://www.ncbi.nlm.nih.gov/geo/, GSE136909.

## Supplementary material

SUPPLEMENTARY FIGURE S1 A double-tagged iGPA cell line supports HC differentiation **(A)** Above: schematics of constructs used triple-tagged line, double-tagged line (“iGPA” in this work), original untagged line (“iGPA-Myo7a:mVenus”). Below: expression of HC markers (red) in induced EBs relative to mVenus (green) and DAPI (blue). Double tagged-line supports HC marker expression but triple-tagged line does not. **(B)** qPCR in EBs for expression of HC markers. Time course after induction for iGPA-Myo7a:mVenus’ (grey) and double-tagged iGPA (black) cell lines. **(C)** qPCR in EBs for expression of HC markers. Addition of RA increases expression of HC markers in the double-tagged iGPA line.

SUPPLEMENTARY FIGURE S2 Transcriptomes from iGPA and iAtoh1 EBs **(A)** Volcano plot of iAtoh1 EBs (Dox induced vs. uninduced); GO analysis of upregulated genes showing nine most significant terms. **(B)** Volcano plot of iGPA EBs (Dox induced vs. uninduced); GO analysis of upregulated genes. **(C)** Cluster analysis of genes upregulated in iAtoh1 cells (cluster 1), divided according to presence or absence of expression in iGPA cells (clusters 1A and 1B). The genes in cluster 1B (genes expressed in iAtoh1 but not iGPA) are shown expanded below.

SUPPLEMENTARY FIGURE S3 Coexpression of Atoh1 and Pou4f3 (iP+A cell line) induces sensory but not HC genes **(A)** qPCR in EBs of iAto1, iGPA and iP+A for HC (Cdh23, Otof, Lhx3, Myo7a, Espin, Myo6) and neuronal (Tubb3, Barhl2, Dcx) markers. **(B)** Cluster analysis of transcriptomes for iAtoh1 and iP+A EBs.

SUPPLEMENTARY FIGURE S4 Atoh1 genomic binding sites in iAtoh1 and iGPA cells **(A)** Venn diagram of Atoh1 genomic binding sites in iAtoh1 and iGPA cells. **(B)** Atoh1 target genes shared between iAtoh1 and iGPA cells. Examples of genes differentially expressed in both iAtoh1 and iGPA cells that also share Atoh1 binding peaks.

SUPPLEMENTARY FIGURE S5 DNA motifs in Atoh1 binding peaks **(A)** Number of E box motifs in binding peaks associated with the three classes of Atoh1 binding site in Figure 4A. **(B)** *de novo* sequence motifs enriched in the three classes of Atoh1 binding peaks. E box motifs are detected in all classes and Pou4f motifs are detected in the iGPA-unique peaks. Gfi1 motifs are not detected at all.

SUPPLEMENTARY FIGURE S6 Direct binding of Atoh1 and Pou4f3 to a subset of genomic locations **(A)** *de novo* sequence motifs enriched in Atoh1/Pou4f3 co-bound peaks from iGPA cells (middle) compared with peaks bound only by Atoh1 (upper) or Pou4f3 (lower). **(B)** Atoh1 and Pou4f DNA motifs associated with Atoh1 and Pou4f3 binding peaks.

SUPPLEMENTARY FIGURE S7 Gfi1 has both positive and negative effects on Atoh1-induced transcriptomes **(A)** Cluster analysis of gene expression in iGfi1, iAtoh1, iG+A, and iGPA cells. Group 4 (Atoh1-dependent genes that are repressed by presence of Gfi1) is shown expanded to the right. **(B)** Examples of Gfi1 binding peaks at Gfi1-repressed genes.
