# Supplementary figures and images for: Repurposing the lineage-determining transcription factor Atoh1 without redistributing its genomic binding sites
Source: Front Cell Dev Biol. 2022 Nov 7;10:1016367. doi: 10.3389/fcell.2022.1016367 (PMC9676683; doi:10.3389/fcell.2022.1016367)

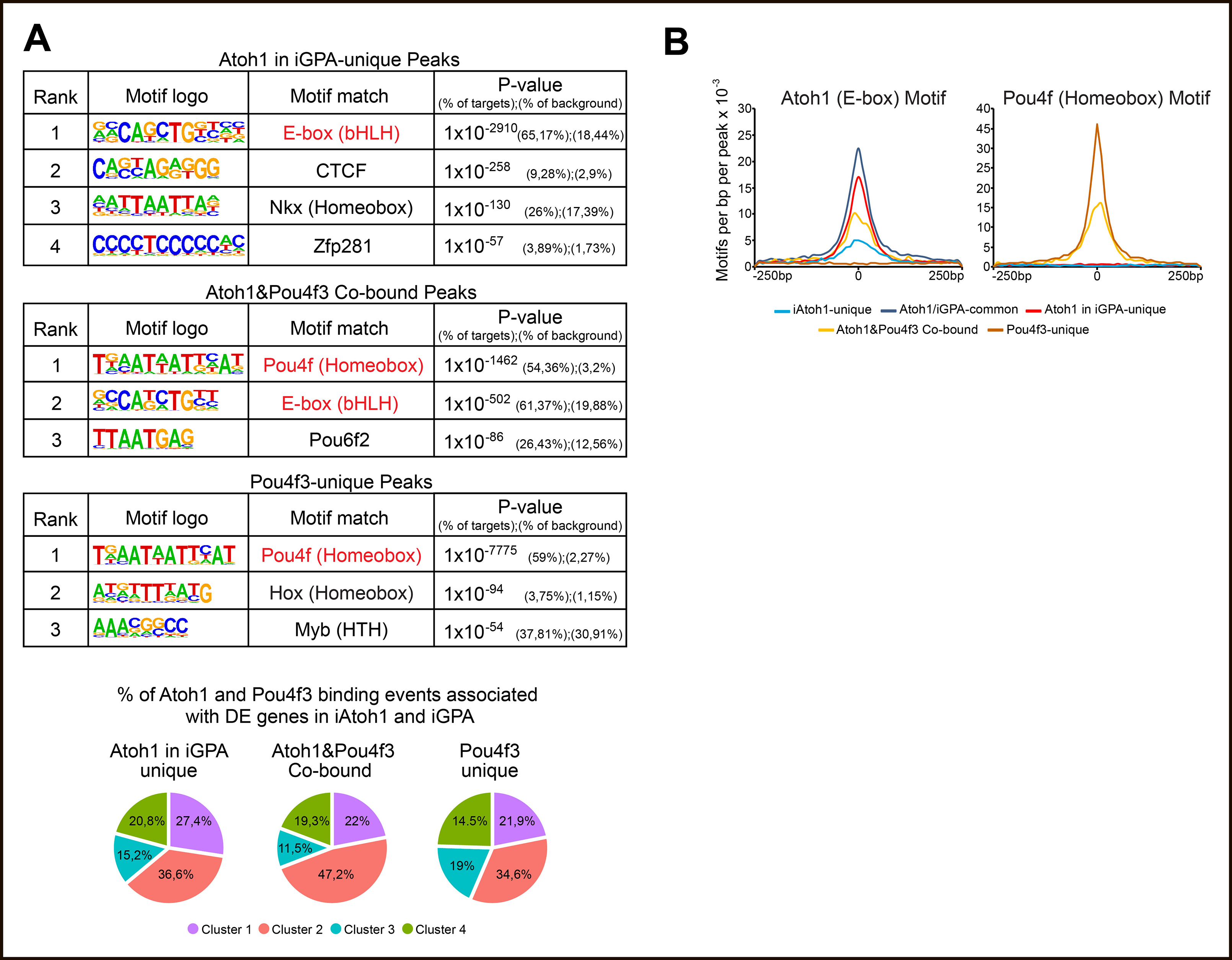

Supplement: Supplementary file 1 [file Image6.TIF]

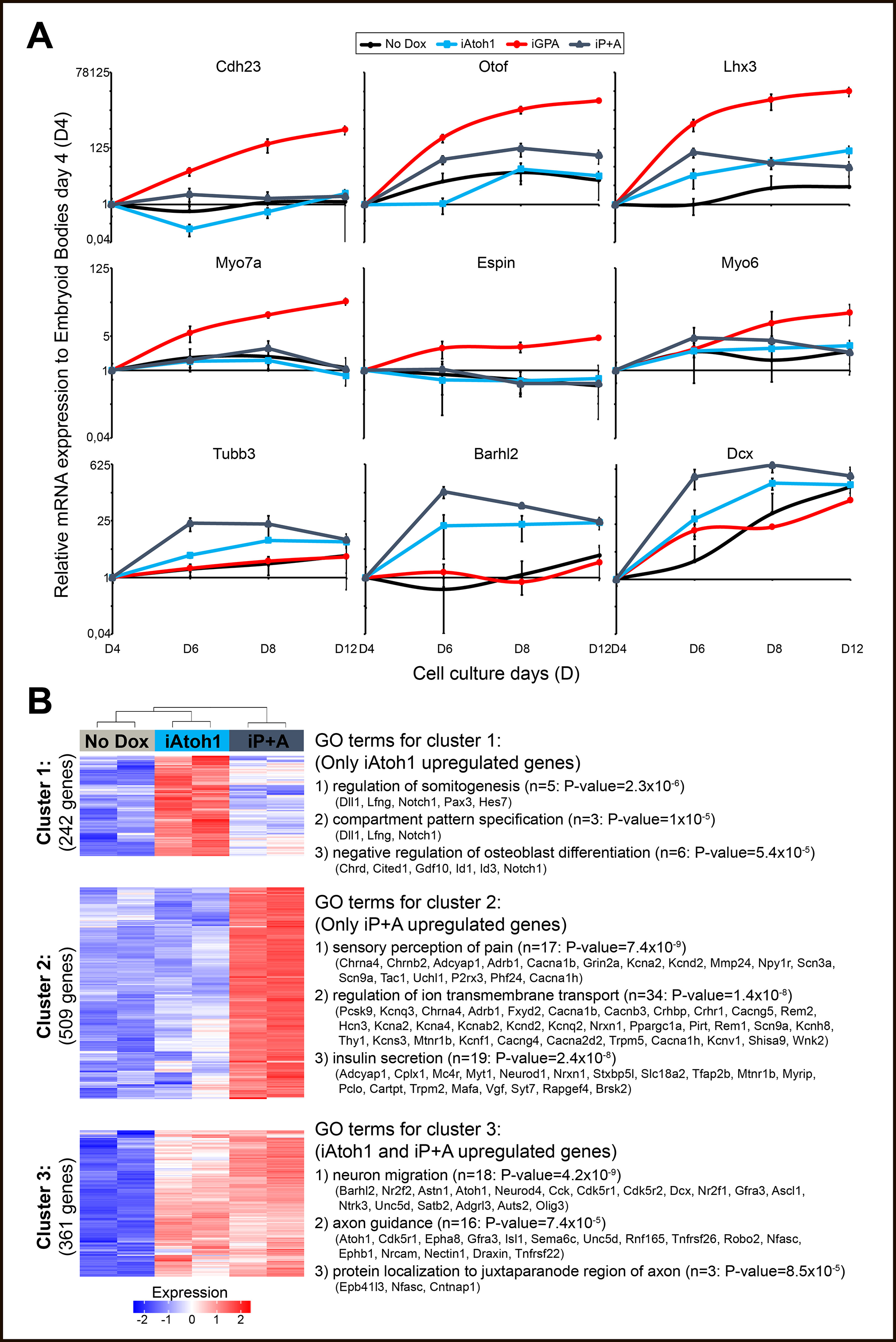

Supplement: Supplementary file 2 [file Image3.TIF]

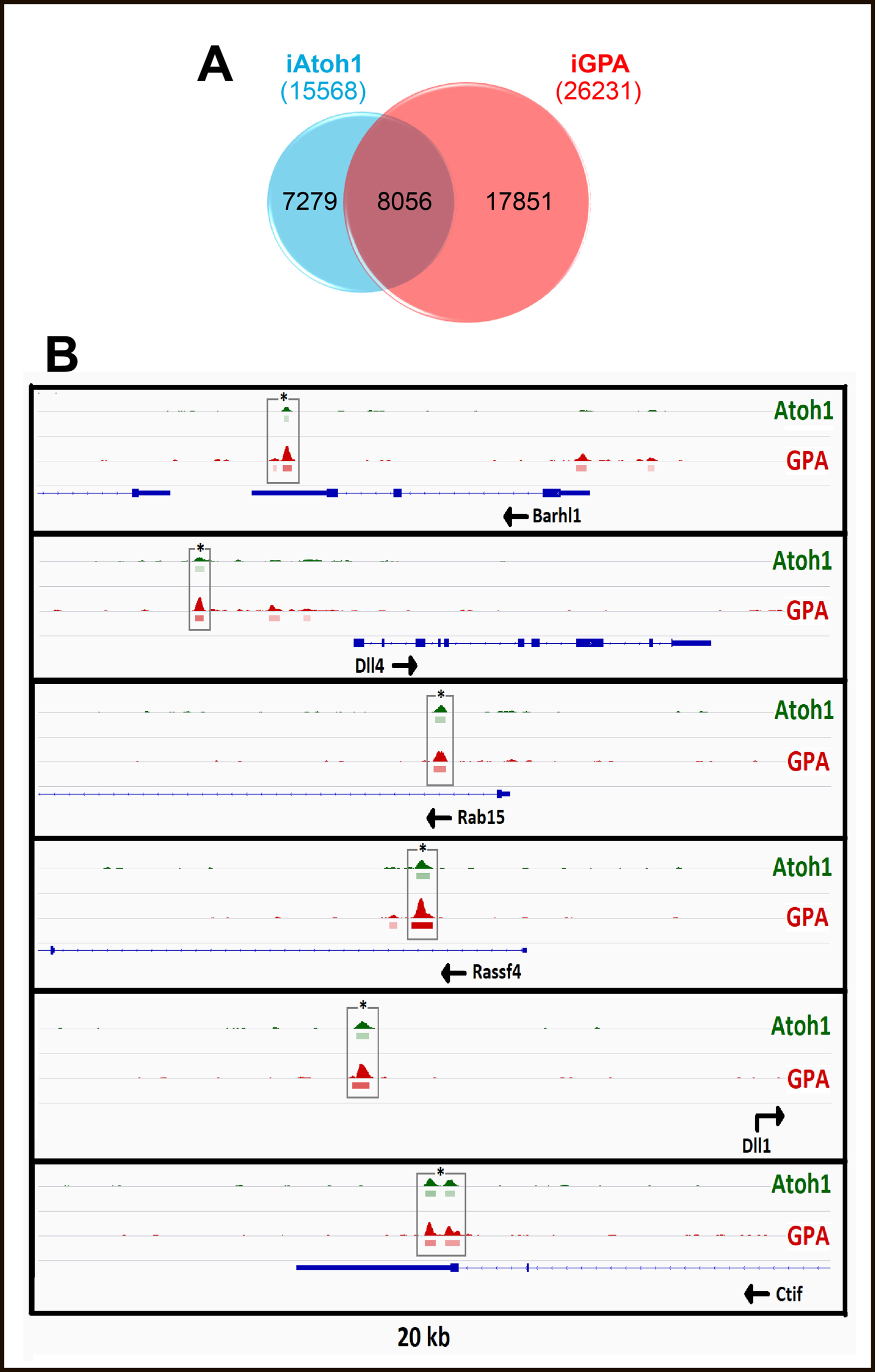

Supplement: Supplementary file 3 [file Image4.TIF]

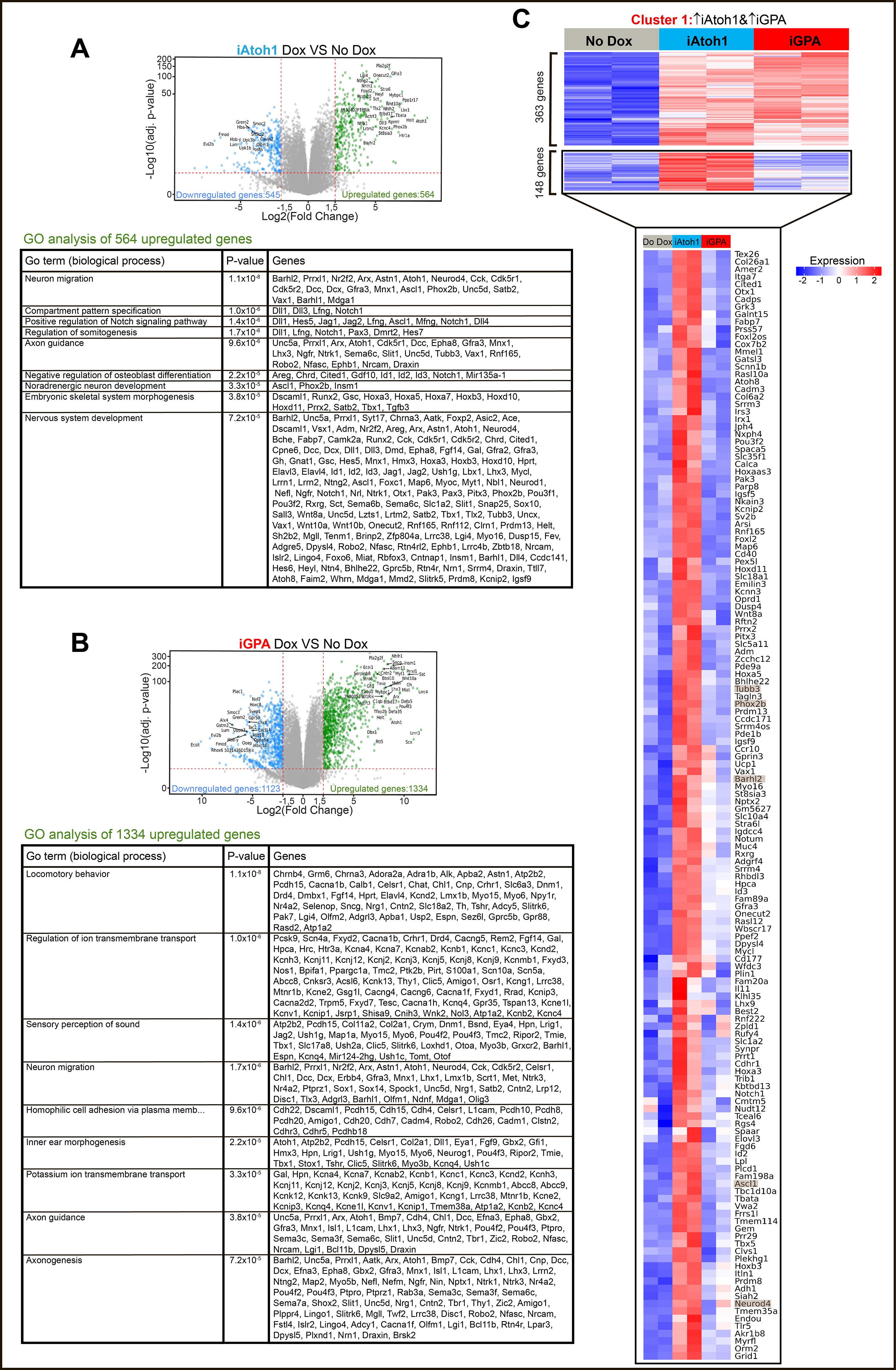

Supplement: Supplementary file 4 [file Image2.TIF]

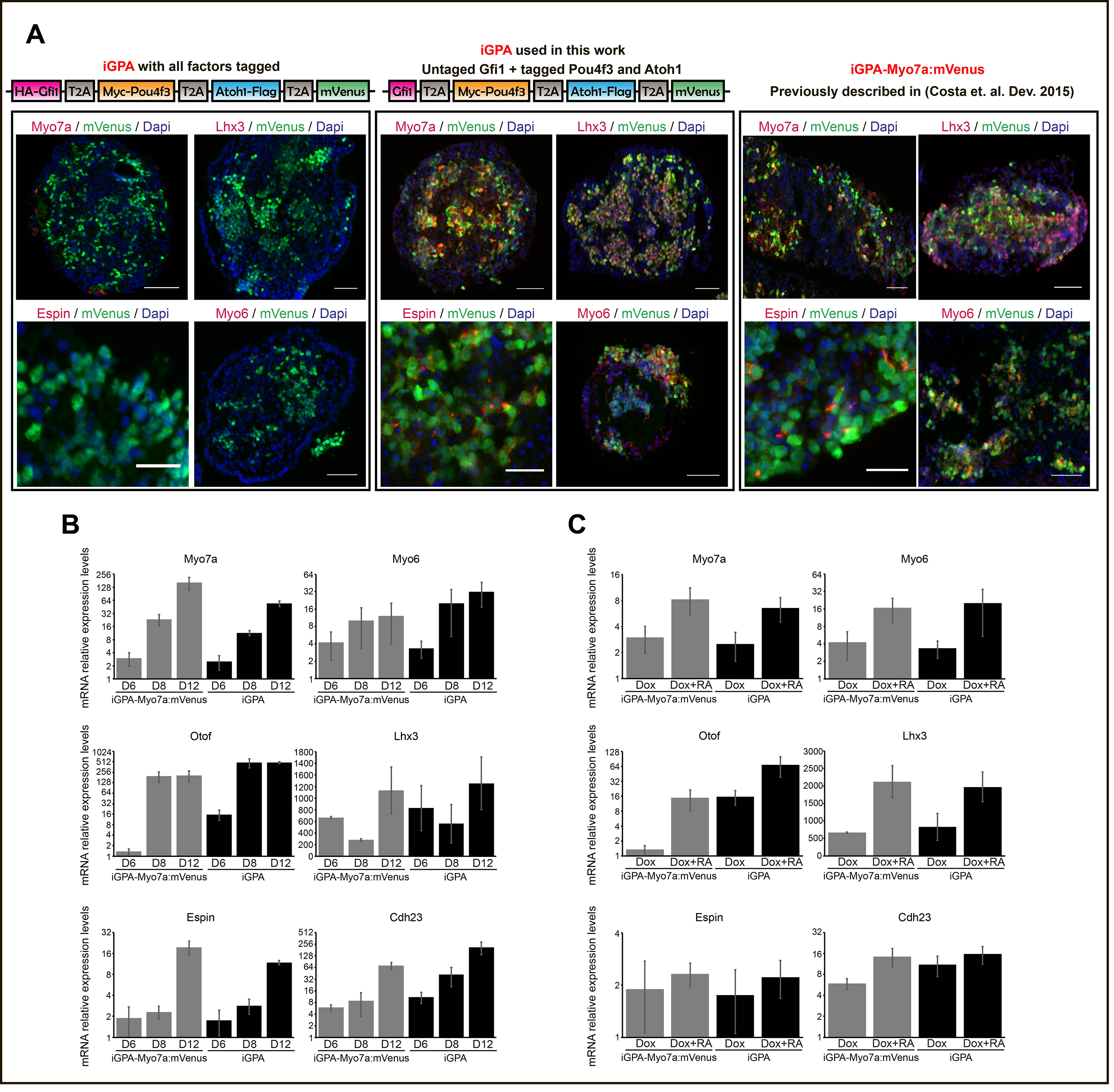

Supplement: Supplementary file 5 [file Image1.TIF]

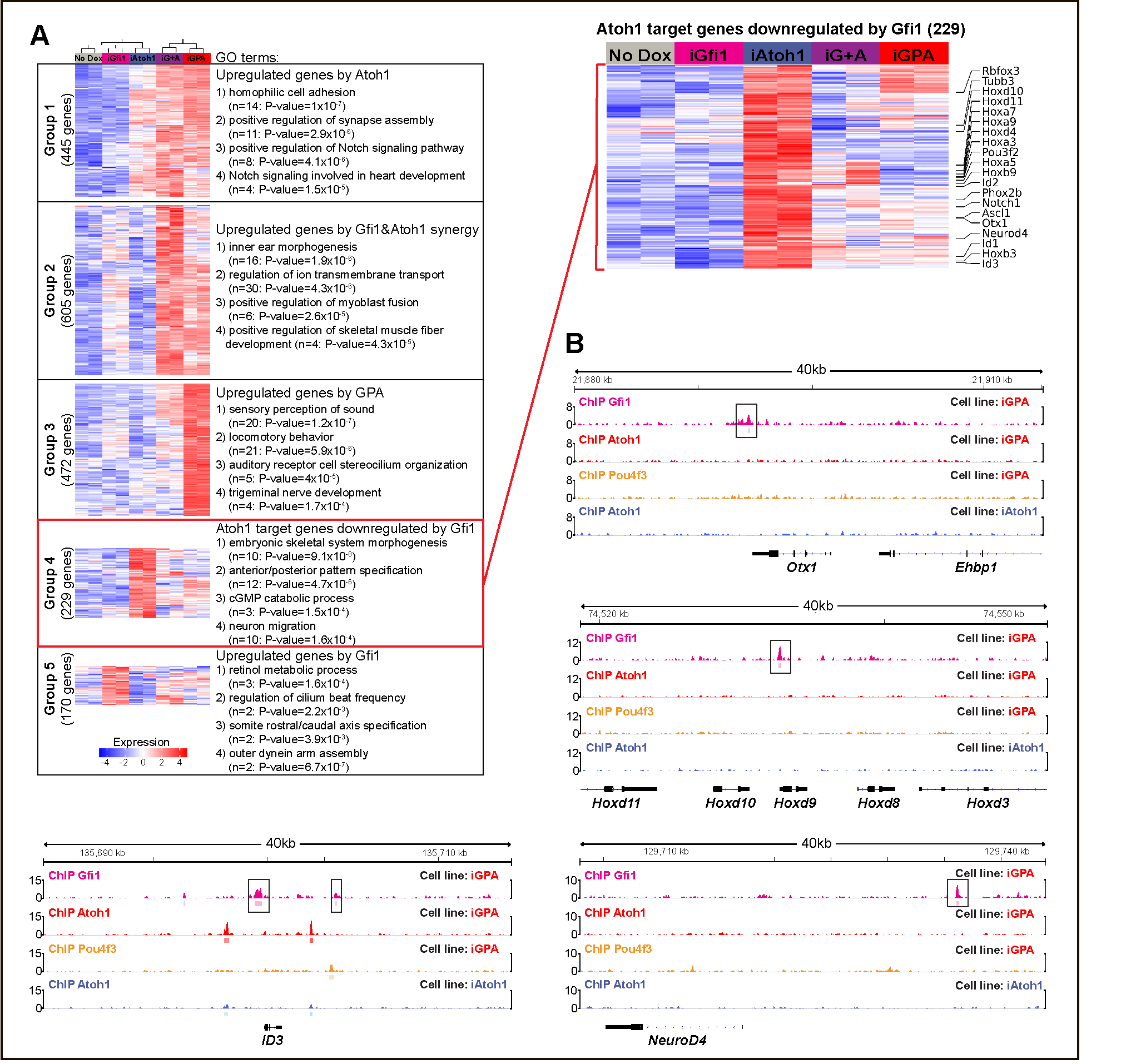

Supplement: Supplementary file 6 [file Image7.TIF]

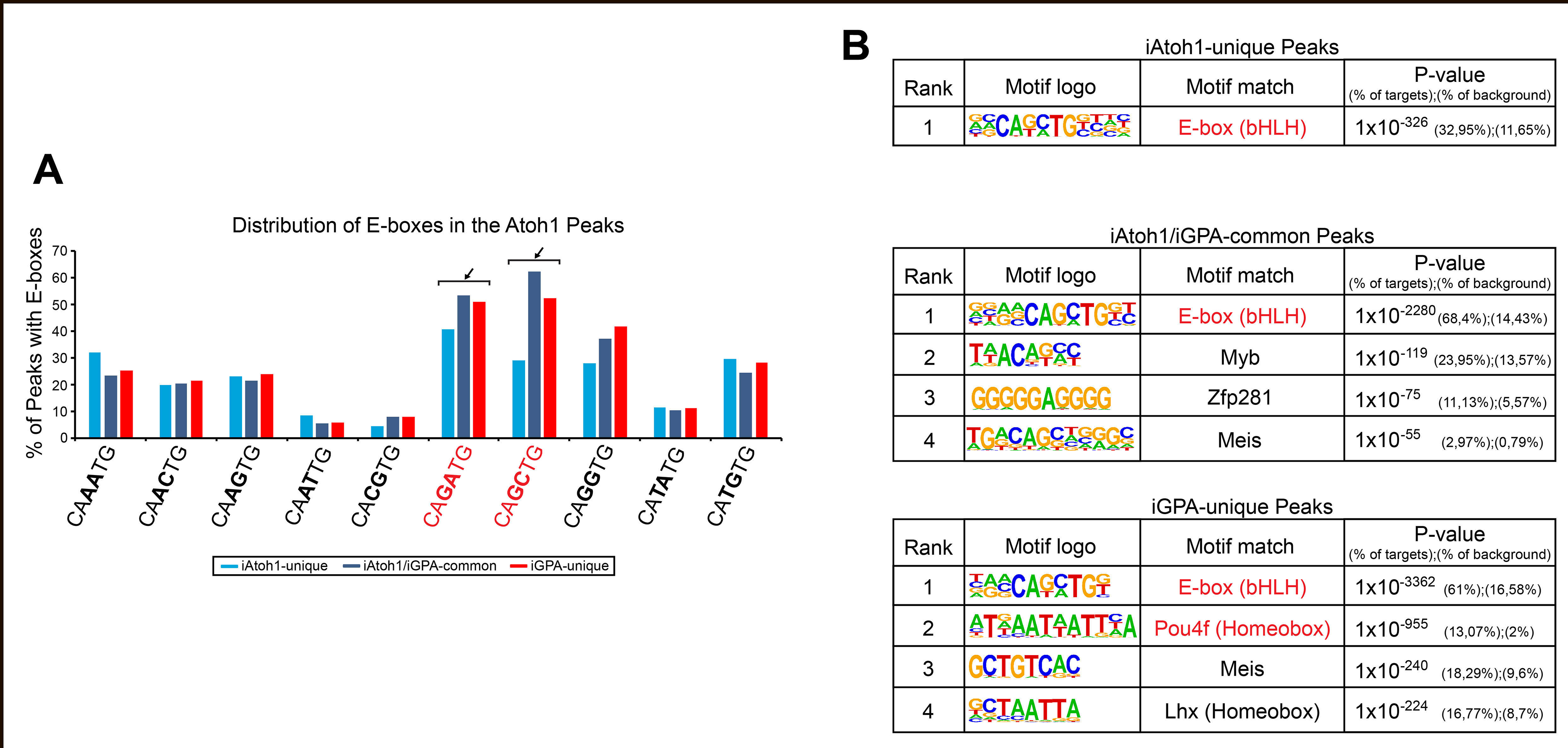

Supplement: Supplementary file 8 [file Image5.TIF]
